# Resonance Analysis as a Tool for Characterizing Functional Division of Layer 5 Pyramidal Neurons

**DOI:** 10.3389/fncom.2018.00029

**Published:** 2018-05-03

**Authors:** Melvin A. Felton Jr., Alfred B. Yu, David L. Boothe, Kelvin S. Oie, Piotr J. Franaszczuk

**Affiliations:** ^1^Computational and Information Sciences Directorate, U. S. Army Research Laboratory, Adelphi, MD, United States; ^2^Human Research and Engineering Directorate, U. S. Army Research Laboratory, Adelphi, MD, United States; ^3^Department of Neurology, Johns Hopkins University School of Medicine, Baltimore, MD, United States

**Keywords:** resonance, layer 5 pyramidal neuron, functional division, electrical coupling, association, ionic conductance

## Abstract

Evidence suggests that layer 5 pyramidal neurons can be divided into functional zones with unique afferent connectivity and membrane characteristics that allow for post-synaptic integration of feedforward and feedback inputs. To assess the existence of these zones and their interaction, we characterized the resonance properties of a biophysically-realistic compartmental model of a neocortical layer 5 pyramidal neuron. Consistent with recently published theoretical and empirical findings, our model was configured to have a “hot zone” in distal apical dendrite and apical tuft where both high- and low-threshold Ca^2+^ ionic conductances had densities 1–2 orders of magnitude higher than anywhere else in the apical dendrite. We simulated injection of broad spectrum sinusoidal currents with linearly increasing frequency to calculate the input impedance of individual compartments, the transfer impedance between the soma and key compartments within the dendritic tree, and a dimensionless term we introduce called resonance quality. We show that input resonance analysis distinguished at least four distinct zones within the model based on properties of their frequency preferences: basal dendrite which displayed little resonance; soma/proximal apical dendrite which displayed resonance at 5–23 Hz, strongest at 5–10 Hz and hyperpolarized/resting membrane potentials; distal apical dendrite which displayed resonance at 8–19 Hz, strongest at 10 Hz and depolarized membrane potentials; and apical tuft which displayed a weak resonance largely between 8 and 10 Hz across a wide range of membrane potentials. Transfer resonance analysis revealed that changes in subthreshold electrical coupling were found to modulate the transfer resonant frequency of signals transmitted from distal apical dendrite and apical tuft to the soma, which would impact the frequencies that individual neurons are expected to respond to and reinforce. Furthermore, eliminating the hot zone was found to reduce amplification of resonance within the model, which contributes to reduced excitability when perisomatic and distal apical regions receive coincident stimulating current injections. These results indicate that the interactions between different functional zones should be considered in a more complete understanding of neuronal integration. Resonance analysis may therefore be a useful tool for assessing the integration of inputs across the entire neuronal membrane.

## Introduction

Neocortical architecture facilitates association-based information processing where feedforward and feedback signals connect the many different processing stages of the neocortex. The associative nature of neocortical function can even be observed on the scale of single neurons, such as layer 5 pyramidal neurons that play a central role in the functioning of neocortical microcircuits (Larkum et al., 1999; Larkum, 2013). The neuronal membrane of these neurons has a large spatial extent and is usually spread throughout all neocortical layers, and different parts of the neuron (e.g., proximal vs. distal) receive inputs from different regions of the brain (Larkum et al., 2009). Direct feedforward projections, which are typically of local origin, synapse close to the soma on proximal dendrites; on the other hand, feedback projections, which tend to originate from far away sources, like non-specific thalamocortical neurons or distant cortical neurons, synapse on the distal regions of the apical dendrite and apical tuft (Spruston, 2008; Hawkins and Ahmad, 2016). The perisomatic and distal apical regions of layer 5 pyramidal neurons have been identified as two distinct “zones” that both mediate action potential initiation (Larkum, 2013). Moreover, because these two spike initiation zones are electrically coupled, the pyramidal neuron is able to detect coincident feedforward input to its perisomatic regions and feedback input to its distal apical regions.

The concept of distinct functionally-defined zones has been expanded by taking into account additional functional aspects, such as the processes of synaptic integration within the large and complex membrane of layer 5 pyramidal neurons (Williams and Stuart, 2003; Polsky et al., 2004; Spruston and Kath, 2004). For instance, Spruston and Kath (2004), proposed a three-layer model of synaptic integration where: (1) basal/oblique dendrites and apical tuft are two distinct zones that collectively comprise an input layer, (2) proximal apical dendrite/soma and distal apical dendrite are two distinct zones that collectively comprise an integration layer, and (3) the axon hillock itself constitutes an output layer.

Previously, resonance analysis has been shown to be a useful tool, both experimentally and computationally, for distinguishing and defining functional zones (Hutcheon and Yarom, 2000; Izhikevich et al., 2003; Nusser, 2009; Zhuchkova et al., 2013). That is, non-uniform ionic conductance expression throughout the neuronal membrane can establish distinct regions that differ in terms of their frequency preference to subthreshold oscillatory input. Therefore, in biologically-realistic compartmental models of pyramidal neurons, the particular ionic conductances that are defined for a given model compartment largely determines the resonant properties of that compartment (Reyes, 2001; Lörincz et al., 2002). Differences in compartment resonant properties can, in turn, be interpreted in terms of different functional roles. Hu et al. (2009), used resonance analysis on CA1 hippocampal pyramidal neuron models (as well as experimentally) and identified two complementary resonances (roughly 3–12 Hz), each generated by distinct mechanisms, for signals transmitted to-and-from the soma and distal apical dendrite when membrane potentials are below −55 mV.

In the current study, we employ a resonance-based computational approach to studying the spatial distribution of frequency preference within a realistic layer 5 pyramidal neuron model. We began with a model whose post-synaptic responses have been tuned to generate action potentials in response to simultaneous input to distal apical regions and the soma to simulate the associative function of these neurons. We examined the resonance responses of this model to oscillatory input in biologically relevant frequency ranges (1–40 Hz). We then used transfer resonance to examine the electrical coupling that occurs between distinct regions of the model via both subthreshold oscillations and the generation of both perisomatic and distal apical action potentials. We find that tuning of a neuron's post-synaptic physiological properties to enhance association between feedforward and feedback inputs impacts which frequencies it is expected to respond to most strongly and reinforce.

## Models and analysis

### Model configuration

We used the GEneral NEural SImulation System (GENESIS) environment to construct a model neuron that has properties of neocortical layer 5 pyramidal neurons (Bower and Beeman, 2003). Our model is an adaptation of the regular spiking, tufted layer 5 neuron constructed by Traub et al. (2005) (for morphology, passive electrical properties) and Traub et al. (2003) (for conductance kinetics). We made no changes to morphology. All but two of the ionic channels included in the original model remain throughout our simulations. Specifically, resonance analysis requires that neuronal responses be subthreshold. To assure subthreshold behavior in our resonance simulations, we therefore removed the fast sodium conductance [g_Na(F)_], which produces action potential onset (Hodgkin and Huxley, 1952) and the Ca^2+^ dependent K^+^ afterhyperpolarization current which acts as an integrating current to control bursting behavior (Mainen and Sejnowski, 1996). Furthermore, the kinetics for all but one of the ionic conductances remained the same. The lone adjustment was made to M current [g_K(M)_] kinetics where we shifted activation dynamics up to 15 mV in the hyperpolarized direction near the base of the activation curve, and reducing its time constant maximum value by 40 ms (see Equations S1, S2 and Figure S1). Changing g_K(M)_ in this way allows this channel to become active at subthreshold membrane potentials, which more accurately captures the resonance characteristics attributed to g_K(M)_ in neocortical pyramidal neurons when their membrane potential is between rest and depolarized to 30 mV (Gutfreund et al., 1995; Hutcheon and Yarom, 2000). In addition we updated some conductance density values which have been shown experimentally to diverge from those in the original (Traub et al., 2005) model. A more complete description of the model configuration is found in the Supplementary Materials.

Our model was configured to behave similarly to layer 5b pyramidal neurons that display back propagation-activated Ca^2+^ spikes in distal apical dendrite (Larkum et al., 1999; Hay et al., 2011; Larkum, 2013). We incorporate the concept of a distal apical “hot zone,” which is defined as distal apical dendrite (model compartments 16–18 in Table S1) and apical tuft (model compartments 19) with densities for the high-threshold Ca^2+^ conductance [g_Ca(H)_] and low-threshold Ca^2+^ conductance [g_Ca(L)_] 10 and 100 times higher than anywhere else in the apical dendrite, respectively. This type of configuration has been shown to faithfully reproduce the distal apical dynamics, such as Ca^2+^ spike generation, that are necessary for the coupling of perisomatic and distal apical regions of layer 5b pyramidal neurons (Hay et al., 2011).

### Model behavior

**Figure 1:**
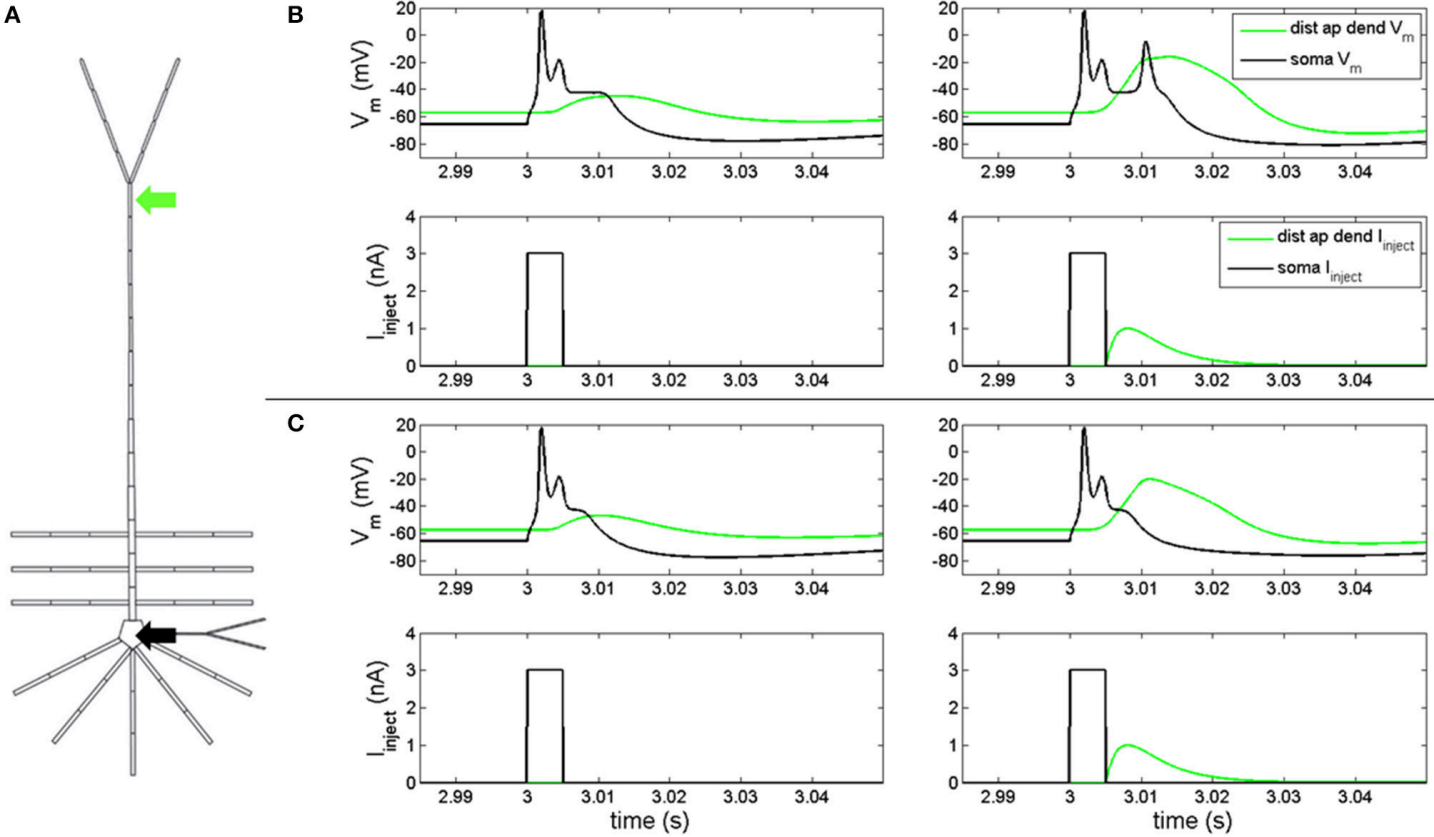
Electrical coupling between distal apical dendrite and soma. **(A)** current injections to distal apical dendrite (green arrow) and soma compartments (black arrow) (neuron rendering from Traub et al., 2005) (**B**, left) model response to 3 nA, 5 ms step current injection into soma when hot zone is in-place. (**B**, right) Model response to 3 nA, 5 ms somatic step current and EPSP-like current injection to distal apical dendrite (1 nA peak, 5 ms delay relative to somatic step current) when hot zone is in-place. (**C**, left) Model response to 3 nA, 5 ms step current injection into soma without hot zone. (**C**, right) Model response to 3 nA, 5 ms somatic step current and EPSP-like current injection to distal apical dendrite (1 nA peak, 5 ms delay relative to somatic step current) without hot zone.

In order to determine if our model reproduced feedforward-feedback interactions, we injected a depolarizing current into the soma and distal apical dendrite compartments (Figure 1A) with g_Na(F)_ active. A 3 nA, 5 ms square pulse injected into the soma of the model with the hot zone (Figure 1B, left-bottom) evoked an action potential spike and spikelet followed by an afterdepolarization potential (Figure 1B, left-top, black line). This somatic depolarization back propagates to distal apical dendrite and leads to depolarization of the local membrane potential (Figure 1B, left-top, green line). When a back propagated pulse evoked by a somatic square wave injection arrives at distal apical dendrite at the same time as a current injection (given by equation S3) into the distal apical dendrite (Figure 1B, right-bottom), a very broad Ca^2+^ spike can be produced in a spike initiation region in the distal apical dendrite (Figure 1B, right-top, green line). This Ca^2+^ spike travels along the apical dendrite to the soma and can cause the soma to depolarize further and emit an additional action potential (Figure 1B, right-top).

Figure 1C illustrates the scenario when the distal apical hot zone has been removed, such that compartments comprising distal apical dendrite (16–18, see Table S1) and apical tuft (19) were given the same density values for g_Ca(H)_ and g_Ca(L)_ as the rest of the apical dendrite (6–15). A 3 nA, 5 ms square pulse injected into soma (Figure 1C, left-bottom) evoked an action potential spike and spikelet followed by an afterdepolarization potential (Figure 1C, left-top, black line) that was reduced in duration relative to the neuron containing the hot zone (Figure 1B, left-top, black line). The somatic depolarization back propagates to distal apical dendrite and leads to a depolarization of the local membrane potential (Figure 1C, left-top, green line) that is also reduced relative to the neuron containing the hot zone (Figure 1B, left-top, green line). When a back propagated pulse evoked by a somatic square wave injection arrives at distal apical dendrite at the same time as a current injection into the distal apical dendrite (Figure 1C, right-bottom), a less robust Ca^2+^ spike is initiated in the distal apical dendrite, indicating a reduction in electrical coupling between distal apical dendrite and soma. The result is that the soma does not depolarize further and does not emit an additional action potential as it did in the case with the distal apical hot zone (compare Figures 1C,B, right-top, black lines).

### Resonance analysis

Resonance analysis was applied on both the model neuron in Figure 1B, the one with a distal apical hot zone, and the model neuron in Figure 1C, the one without a hot zone. In both cases, g_Na(F)_ was removed to prevent action potential generation, as described in section Model Configuration, above. Toggling the hot zone in this manner allowed resonance analysis to be used as a tool to investigate the role of g_Ca(H)_ and g_Ca(L)_ in the coupling of distal apical and perisomatic regions of the model. There were two phases to our resonance analysis: input and transfer.

#### Input resonance analysis

**Figure 2:**
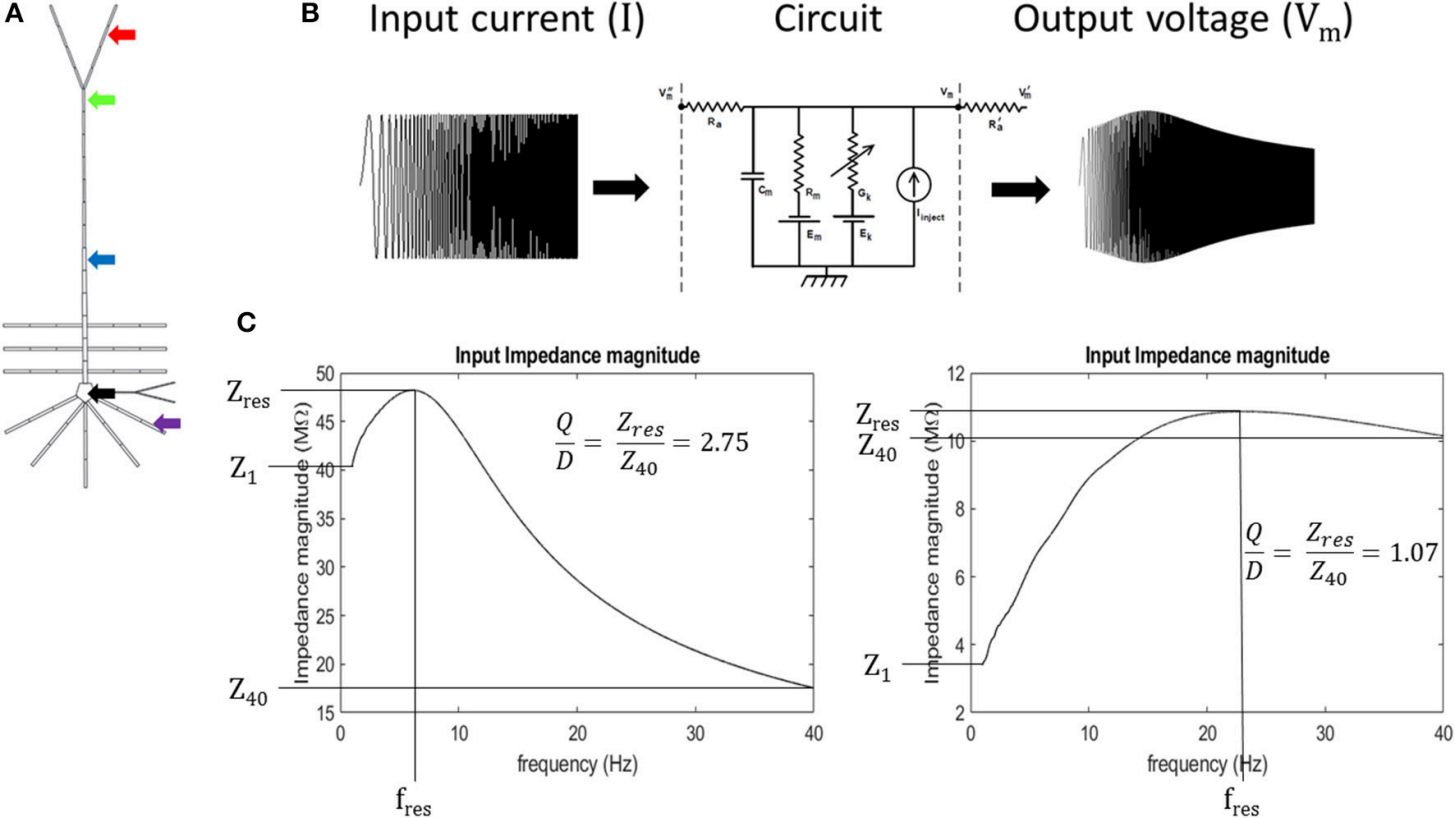
Experiment schematic **(A)** arrows point to compartments used in resonance analysis: black- soma, purple- basal dendrite, blue- middle apical dendrite, green- distal apical dendrite, and red- apical tuft. **(B)** Schematic of circuit response to simulated chirp current injection (circuit diagram from Bower and Beeman, 2003). **(C)** quantification of impedance curve using resonance quality (Q/D). Left—example impedance curve with high Q/D. Right—example impedance curve with low Q/D.

Input resonance analysis was used to examine local frequency preference throughout the model. To characterize the input resonance properties of our model neuron, we systematically injected “chirp” currents, or, broad-spectrum sinusoidal currents with linearly increasing frequency (0–40 Hz over 65 s) into a compartment within the following regions of the model neuron: soma (specifically, compartment 2 in Table S1), basal dendrite (5), middle apical dendrite (11), distal apical dendrite (18), and apical tuft (19) (Figures 2A,B). 40 Hz was set as the maximum frequency for the analysis based on preliminary work where the soma and distal apical dendrite compartments were held at depolarized membrane potentials and injected with a chirp current with linearly increasing frequency from 0 to 200 Hz over 325 s. During these tests, resonance was not observed above 30 Hz, therefore, we report model behavior up to 40 Hz in order to fully capture all resonance observed.

During the input resonance analysis, compartments' membrane potentials were varied from −80 to −30 mV for soma and basal dendrite, and −80 to 0 mV for apical dendrite and apical tuft compartments, in steps of 5 mV using a DC offset current (for DC offset values see Supplementary Materials). These membrane potential ranges were selected based on physiological considerations to avoid evaluating membrane resonance during significant refractory periods. For instance, the maximum membrane potential for the soma and basal dendrite compartments was selected based on the peak value of the somatic afterdepolarization potential after the neuron has fired an action potential; the maximum membrane potential for apical dendrite and tuft compartments were selected based on peak membrane potential after local current injection is timed to match the arrival of a back propagated somatic action potential (see Figure 1B, right-top).

At each of these membrane potentials, a chirp current was injected into the compartments and we calculated the input impedance by dividing the Fourier spectrum of the resultant membrane potential by the Fourier spectrum of the chirp current, F_Vm_ and F_I_, respectively (Equation 1). Chirp current amplitudes were chosen that kept the resultant membrane potential oscillations below approximately 8 mV in peak-to-peak amplitude for each compartment. This ensures that sufficiently non-overlapping regions of the activation curves for the ionic conductances in the model are examined. The chirp current amplitudes were as follows: 10 pA, basal dendrite; 50 pA, apical tuft; 75 pA, soma; 100 pA, middle apical dendrite; and 115 pA, distal apical dendrite. We obtained our Fourier spectrums by applying a Fast Fourier Transform (FFT) algorithm (Matlab™ FFT function) on model compartment's membrane potential over the 65 s chirp injection epoch and applying 100-pt moving average smoothing in the frequency domain. Impedance was calculated on the interval, 1–40 Hz, to avoid boundary effects associated with applying discrete FFT on a finite sampling window. Resonance quantification analysis was performed on the resultant impedance magnitude curves (simply referred to as impedance in the remainder of the text) by calculating resonance strength (Q), degree of high-pass filtering (D), half-band width (HB), and by identifying the resonant frequency (f_res_) and resonant impedance (Z_res_) value (Erchova et al., 2004) (Equations 2, 3; Figure S8).

We introduce a dimensionless quantity that we call “resonance quality” defined as the ratio of resonance strength and degree of high-pass filtering (Q/D). We used resonance quality as the primary metric to quantify the shape of impedance curves within the 1–40 Hz interval (Equation 4). It is advantageous to use Q/D to mitigate “false positive” resonance cases when Q is roughly equal to D (Q/D ≈ 1), a condition that is more indicative of broadband or high-pass filtering/amplification as opposed to a well-defined resonance. On the other hand, larger values of Q/D are indicative of well-defined band-pass filtering (large amplitude and narrow peak in impedance curve). Mitigating for false positives becomes important when a compartment passes through its resonant regime as its membrane becomes increasingly depolarized. It was observed during preliminary tests that resonance tends to give way to high-pass filtering in some simulations, particularly for the perisomatic regions of the model. Therefore, Q/D is a metric that accurately captures resonance features of our model on the 1–40 Hz interval. Expressions for our resonance quantification analysis are given in Equations 1–4:

(1) $$\begin{array}{r} Z=\;\frac{F_{Vm}}{F_{I}} \end{array}$$

(2) $$\begin{array}{r} Q=\;\frac{Z_{res}}{Z_{1}} \end{array}$$

(3) $$\begin{array}{r} D=\;\frac{Z_{40}}{Z_{1}} \end{array}$$

(4) $$\begin{array}{r} resonance\;quality=\;\frac{Q}{D}=\;\frac{Z_{res}}{Z_{40}} \end{array}$$

where *Z, F*_*Vm*_, and *F*_*I*_ are the impedance, Fourier spectrum of output compartment membrane potential, and Fourier spectrum of injected chirp current, respectively; *Z*_*res*_*, Z*_1_, and *Z*_40_ are the resonant impedance, impedance at 1 Hz, and impedance at 40 Hz, respectively (Figure 2C).

#### Transfer resonance analysis

Transfer resonance analysis was used to characterize subthreshold interaction, or electrical coupling, between compartments in our model. Transfer impedance was calculated between each dendritic compartment examined in the input resonance analysis and the soma, as well as the transfer impedance between the soma and distal apical dendrite. Transfer impedance was calculated by dividing the Fourier spectrum of the resultant membrane potential in the transfer, or “receiving,” compartment by the Fourier spectrum of the chirp current injected into the input compartment. Transfer resonance was quantified using the same metrics used in the input resonance analysis. The compartments where the chirp current was injected had their membrane potentials varied across the same range as in the input resonance analysis, but in steps of 10 mV, as opposed to 5 mV, using DC offsets (for DC offset values see Supplementary Materials). In addition, the transfer analysis was repeated multiple times, each time with the transfer compartments held at either hyperpolarized, near rest, or various amounts of depolarized membrane potentials using a second DC offset (see Supplementary Materials).

## Results

### Input resonance analysis on model with hot zone

**Figure 3:**
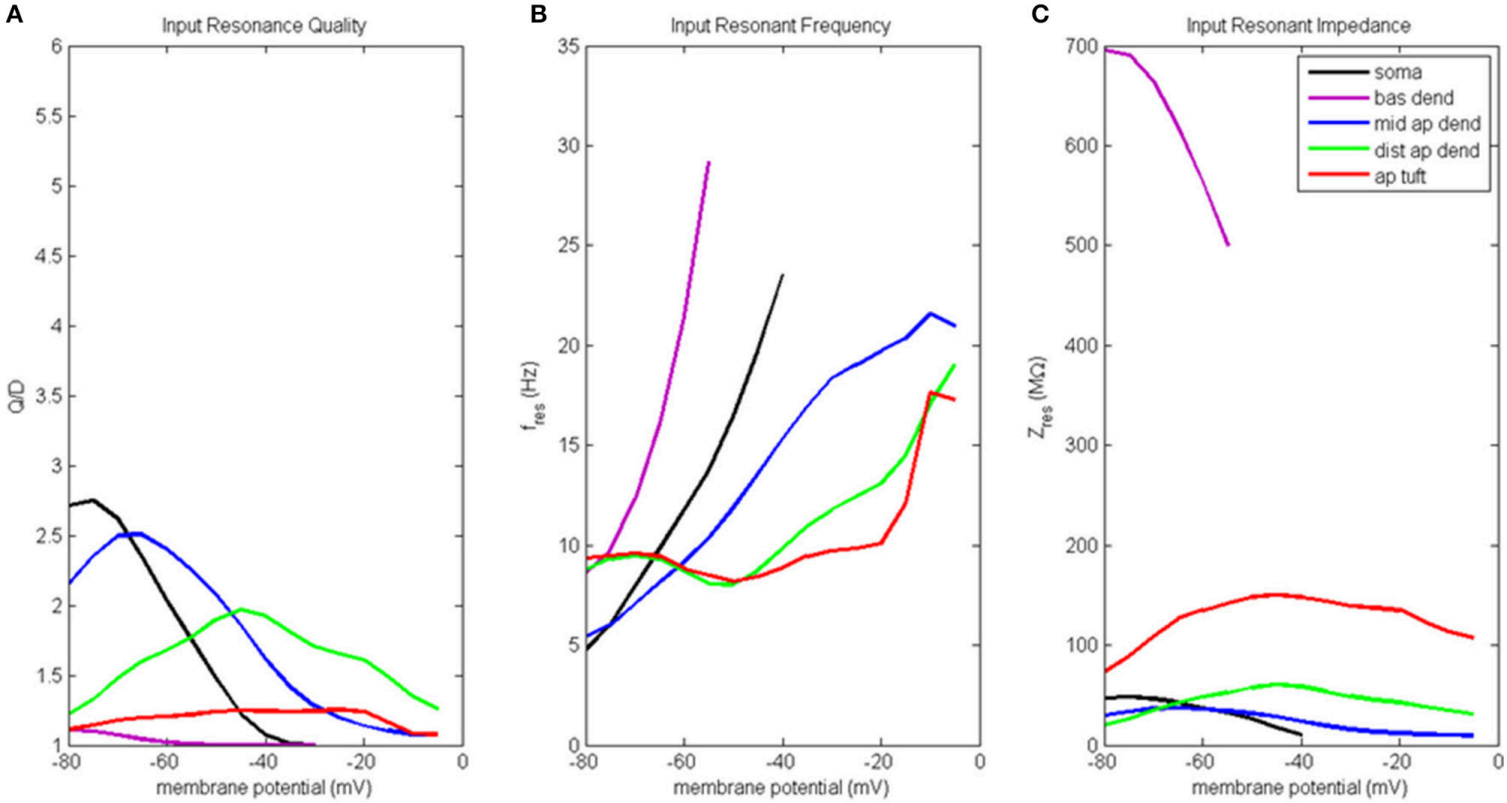
Input resonance analysis. **(A)** Input resonance quality (Q/D), **(B)** input resonant frequency (f_res_), and **(C)** input resonant impedance (Z_res_), vs. initial compartment membrane potential.

Results of the input resonance analysis are shown in Figure 3 (for waveforms of compartment voltage response at key values of membrane potential in different regions of the model neuron, see Figure S3). Profiles of input resonance quality (Q/D) are presented in Figure 3A. For the compartments along the soma-apical dendrite axis, there was a progressive shift in the compartment exhibiting the highest resonance quality as each compartment's membrane potential was increased from hyperpolarized to depolarized potentials. The soma's peak resonance quality, the highest of any compartment, occurred at hyperpolarized membrane potentials. Also note that the soma's resonance quality (Figure 3, black line) reduced to approximately 1 at a membrane potential of around −35 mV; a condition that is not indicative of resonance, but rather of a broadband and/or high-pass impedance curve. Middle apical dendrite resonance quality (blue line) peaked when its membrane potential reached around −65 mV, and it became the compartment with the highest resonance quality. Distal apical dendrite (green line) became the compartment with the highest resonance quality at more depolarized membrane potentials, with a peak at −45 mV, approximately 12 mV above the local resting membrane potential. By contrast, peak resonance quality for apical tuft (red line) occurred around −20 mV, but there was no membrane potential for which apical tuft became the compartment with the highest resonance quality. Basal dendrite (purple line) exhibited only very weak resonance for a small range of membrane potentials, from hyperpolarized potentials up to approximately −55 mV.

In addition to a soma-apical dendrite shift in peak input resonance quality as each compartment's membrane potential was increased from hyperpolarized to depolarized membrane potentials, the input resonance quality curves for the compartments on the soma-apical dendrite axis, including the apical tuft, became progressively flatter the farther the compartment was from the soma. In particular, apical tuft displayed a fairly flat resonance quality curve across almost the entire range of membrane potentials. This progressive flattening of input resonance quality profiles along the soma-apical dendrite axis is similar to the spatial distribution of frequency preference specificity as reflected in the half-bandwidth (HB) of the compartments' impedance curves (Table S2A). The soma and middle apical dendrite have narrow HB at hyperpolarized and resting membrane potentials but their HB widens rapidly with depolarization. On the other hand, distal apical dendrite and apical tuft generally have wider HB than soma or middle apical dendrite. However, the HB of these compartments does become narrower for intermediate levels of depolarization (by up to 9 Hz in some cases for distal apical dendrite).

Input resonant frequency profiles for the compartments in this analysis are shown in Figure 3B. Soma, basal dendrite, and middle apical dendrite all consistently showed rising resonant frequency as membrane potential increased from hyperpolarized to higher levels of depolarization. Somatic resonant frequency increased from 5 Hz at hyperpolarized potentials to 23.5 Hz at −40 mV, the highest membrane potential for which the soma exhibited resonance. Basal dendrite exhibited resonance across an even shorter range of membrane potentials where it sweeps out a wide range of resonant frequencies from 8.5 to 29 Hz. Middle apical dendrite had a resonant frequency profile similar to the soma but with a slower rate of increase. Distal apical dendrite and apical tuft had relatively flat resonant frequency curves, centered around 8–9.5 Hz from hyperpolarized to near resting membrane potentials. The resonant frequency curves for these compartments ultimately increased to > 15 Hz for highly depolarized membrane potentials. Note that at −50 mV, distal apical dendrite began to display a resonant frequency that progressively increased with increasing membrane potentials, much like the soma, basal dendrite, and middle apical dendrite compartments. On the other hand, apical tuft remained relatively flat out to −20 mV, then rapidly increased with further membrane depolarization.

Input resonant impedance profiles for this analysis are shown in Figure 3C. The compartment that showed the highest resonant impedance values was the basal dendrite. Its resonant impedance was 700 MΩ when its membrane potential was −80 mV and rapidly decreased to 500 MΩ as its membrane potential increased to −55 mV. The maximum resonant impedance values for the apical dendrite and apical tuft compartments showed an inverse relationship to these compartments' maximum values in their resonance quality profiles, namely, the highest resonant impedance values belonged to the apical tuft compartment across the entire range of membrane potentials (maximum value 150 MΩ at −45 mV), while the distal apical dendrite was generally greater than middle apical dendrite, except below −70 mV. Distal apical dendrite resonant impedance maximum was 60 MΩ at −45 mV while middle apical dendrite resonant impedance maximum was 37 MΩ at −65 mV. The soma's resonant impedance profile peaked at hyperpolarized potentials (50 MΩ at −75 mV) then decreased as membrane potentials increased to −40 mV.

### Transfer resonance analysis on model with hot zone

#### Soma and distal apical compartments

**Figure 4:**
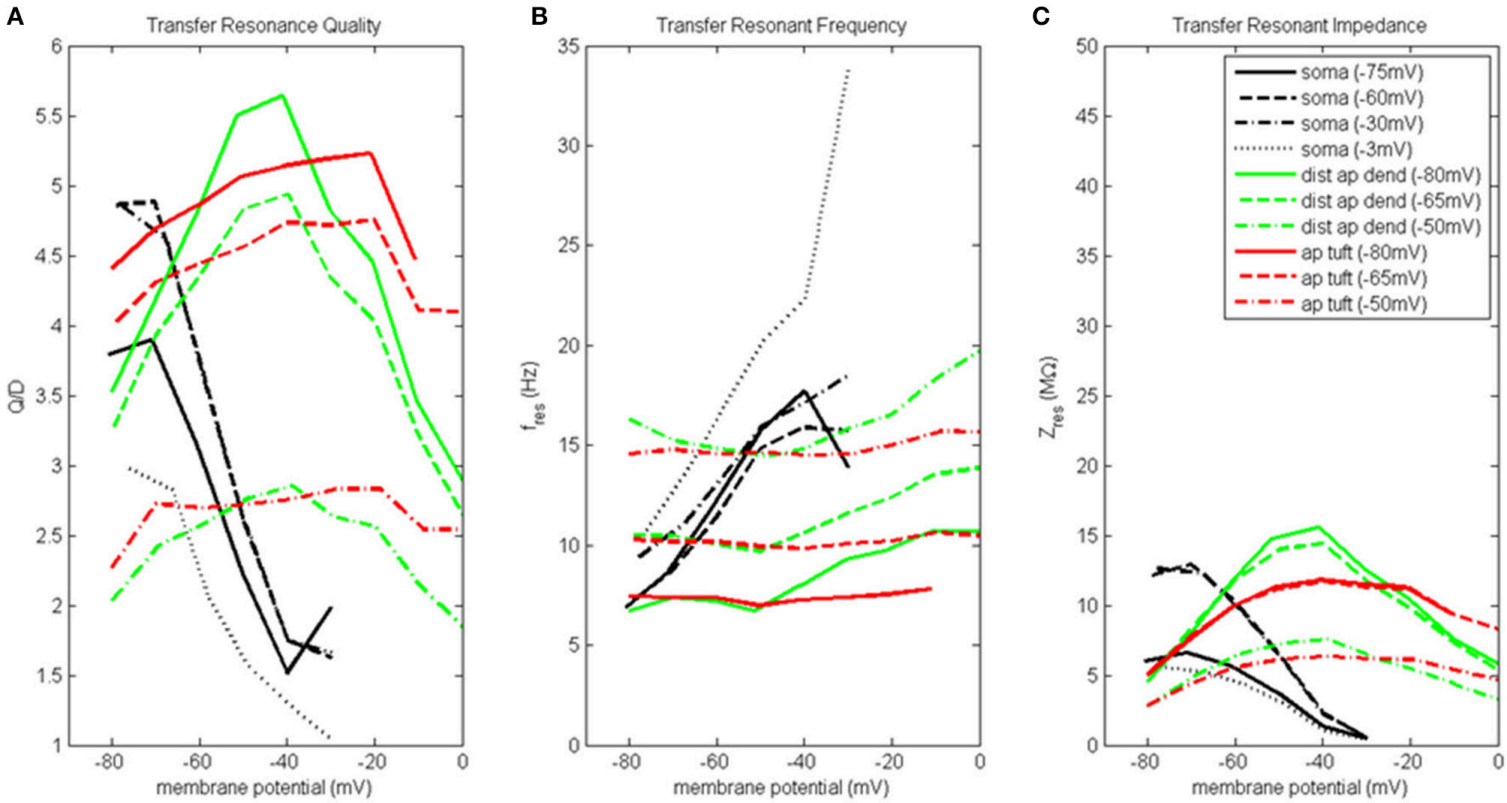
Transfer resonance analysis (soma, distal apical dendrite, apical tuft). **(A)** Transfer resonance quality (Q/D), **(B)** transfer resonant frequency, and **(C)** transfer resonant impedance, vs. initial compartment membrane potential. In the legend, the membrane potential of the transfer compartment is given in parentheses next to the name of the injection compartment.

Transfer resonance analysis was used to examine subthreshold electrical coupling between the dendritic compartments involved in the input resonance analysis and the soma, and between the soma and distal apical dendrite. Because our baseline model neuron is configured to exhibit enhanced spiking due to coupling of the soma and a distal apical spiking zone, we chose to present the results of transfer resonance analysis for the soma and the two distal apical compartments together (Figure 4, Figure S4). The black curves represent the scenarios in which a chirp current was injected into the soma and the resultant membrane potential in the distal apical dendrite was measured. The green curves represent the scenarios in which a chirp current was injected into distal apical dendrite and the resultant membrane potential of the soma was measured. The red curves represent the scenarios in which a chirp current was injected into apical tuft and the resultant membrane potential of the soma was measured. Multiple experimental runs were performed in which the injection compartment membrane potential was stepped through a range of values in steps of 10 mV while the transfer compartment was held at a few select membrane potentials provided in parentheses next to the name of the injection compartment in the legend of Figure 4.

Figure 4A presents the transfer resonance quality profiles for the soma and distal apical compartments. The somatic membrane potentials that resulted in the highest resonance quality between soma and distal apical dendrite were hyperpolarized/resting potentials. As the soma was depolarized above its resting membrane potential, transfer resonance quality decreased rapidly. The highest observed values of resonance quality between soma and distal apical dendrite (Q/D between 4.5 and 5) occurred both when the distal apical dendrite membrane potential was near rest and depolarized to −30 mV. The resonance quality profile for soma-to-distal apical dendrite transmission remains qualitatively the same but is shifted downward when distal apical dendrite membrane potential is hyperpolarized to −75 mV (values between 3.5 and 4 when soma at hyperpolarized/resting membrane potentials) or depolarized further to −3 mV (values between 2.5 and 3 when soma at hyperpolarized/resting membrane potentials).

The membrane potentials of distal apical dendrite that resulted in the highest transfer resonance quality with the soma were between −50 and −40 mV (Figure 4A). As distal apical dendrite membrane potential became more hyperpolarized or more depolarized than these values, transfer resonance quality decreased. The highest observed values of transfer resonance quality between distal apical dendrite and soma (Q/D approximately 5.5) occurred when the somatic membrane potential was hyperpolarized at −80 mV. The resonance quality profile for distal apical dendrite-to-soma transmissions qualitatively remains the same, but is shifted downward when the soma becomes more and more depolarized—peak values between 4.5 and 5 when soma at −65 mV, and peak values between 2.5 and 3 when soma at −50 mV.

The largest transfer resonance quality observed for signals transmitted from apical tuft to soma also occurred when the soma was hyperpolarized and at rest (Figure 4A). Each of these profiles contain values > 4, and their peaks (5.2 and 4.8, respectively) occurred when the apical tuft compartment was at −20 mV. The peak value in the profile for signals transmitted from apical tuft to soma when soma is depolarized to −50 mV reduced to 2.8.

Figure 4B shows the transfer resonant frequency between the soma and distal apical compartments. Transfer resonant frequency from soma to distal apical dendrite increases almost linearly as soma membrane potential is depolarized. When distal apical dendrite membrane potential is between −75 and −30 mV, the resonant frequency profiles of soma-to-distal apical dendrite transmission group together and vary from 7 Hz for hyperpolarized soma (distal apical dendrite at −75 or −60 mV) to 18 Hz for soma depolarized to −30 mV (distal apical dendrite at −30 mV). The highest resonant frequencies for transmission from soma to distal apical dendrite occurred when distal apical dendrite is highly depolarized to −3 mV. Under this condition, the range of somatic transfer resonant frequencies as the soma's membrane potential was varied is 11–34 Hz. However, it should be noted that the frequencies under the condition of both high somatic and distal apical dendrite depolarization are associated with very low resonance quality and therefore represent a very weak frequency preference.

Transfer resonant frequency from distal apical dendrite to soma displayed a banded structure that is based on somatic membrane potential (Figure 4B). The band with the slowest frequencies (roughly 7–11 Hz) occurred when the soma was hyperpolarized; a band with intermediate frequency values (10–14 Hz) occurred when soma was at −65 mV; lastly, a band with the fastest frequencies (14–20 Hz) occurred when soma was depolarized to −50 mV. On the other hand, each of the transfer resonant frequency curves for apical tuft-to-soma transmissions were flatter and non-overlapping. These bands were situated at 7, 10, and 14.5 Hz for hyperpolarized, resting, and depolarized soma, respectively. The bands in resonant frequency for transmission from distal apical dendrite and apical tuft to soma are inversely related to the resonance quality of transmission from distal apical dendrite and apical tuft to the soma, such that the faster the frequency, the lower the transfer resonance quality.

Transfer resonant impedance between soma and distal apical compartments is shown in Figure 4C. Transfer resonant impedance profiles for soma-to-distal apical dendrite transmission and for transmission between both distal apical compartments and the soma are qualitatively similar to their corresponding resonance quality profiles. However, it is easier to identify within the transfer resonant impedance profiles a two-tiered structure in the communication between these two regions of the pyramidal neuron model. For soma-to-distal apical dendrite transmission, the transfer resonant impedance profiles for the cases when distal apical dendrite is at −60 and −30 mV group together, peaking between 13 and 14 MΩ when soma is hyperpolarized, and decreasing rapidly to 0.5 MΩ with somatic depolarization. On the other hand, the transfer resonant impedance profiles for the case when distal apical dendrite membrane potential is hyperpolarized at −75 mV and depolarized strongly to −3 mV group together, with values around 6 MΩ when soma is hyperpolarized and also decreasing to 0.5 MΩ with somatic depolarization.

For distal apical dendrite-to-soma transmission, the transfer resonant impedance profiles for the cases when the soma is at −80 and −65 mV group together, peaking near 15 MΩ when distal apical dendrite is between −50 and −40 mV, and decreasing rapidly to 5 MΩ with either hyperpolarization or depolarization out of this range of dendritic membrane potentials. On the other hand, when the soma is depolarized to −50 mV, the peak in the transfer resonant impedance profile for distal apical dendrite-to-soma transmission is reduced by half to 7.5 MΩ and tapers to 3 MΩ with either hyperpolarization or depolarization out of the −50 to −40 mV range in membrane potential values for distal apical dendrite.

The transfer resonant impedance profiles for apical tuft-to-soma transmissions were generally lower in magnitude, except for compartment membrane potentials > −25 mV, and had a broader shape relative to the profiles for distal apical dendrite. Maximum values for apical tuft-to-soma transmission were 12 MΩ when soma was either hyperpolarized or at rest, while maximum values of the apical tuft-to-soma transfer resonant impedance profile decreased to 6 MΩ when soma was depolarized.

#### Basal dendrite and middle apical dendrite

**Figure 5:**
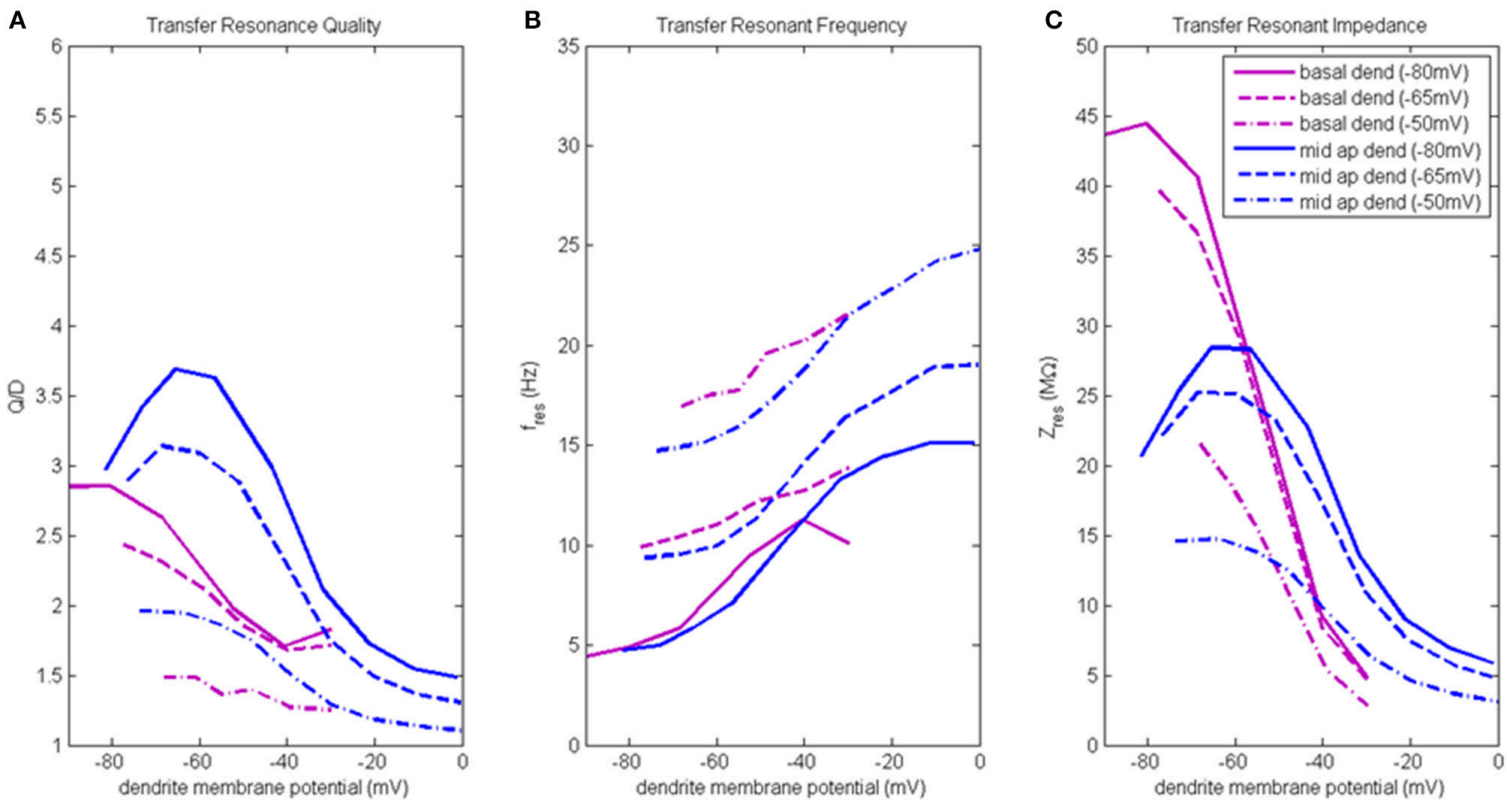
Transfer resonance analysis (basal dendrite and middle apical dendrite). **(A)** Transfer resonance quality (Q/D), **(B)** transfer resonant frequency, and **(C)** transfer resonant impedance, vs. initial compartment membrane potential. In the legend, the membrane potential of the transfer compartment is given in parentheses next to the name of the injection compartment.

Transfer resonance analysis for the other dendritic compartments included in this study are presented in Figure 5 and Figure S4. Each curve was obtained by injecting chirp current into the respective compartment and then measuring the resultant membrane potential in the soma. Once again, there are three profiles per compartment corresponding to the three membrane potential values that the transfer compartment, in this case the soma, was held at–hyperpolarized, resting, and depolarized potentials.

Figure 5A presents the transfer resonance quality profiles for middle apical and basal dendrite compartments (blue and purple lines, respectively). The largest transfer resonance quality observed were for signals transmitted from middle apical dendrite to soma when soma was at hyperpolarized and resting membrane potentials. These values peaked (3.7 and 3.1, respectively) when middle apical dendrite membrane potential was between −70 and −55 mV and then reduce sharply (to 1.5) as the compartment is depolarized. The resonance quality profile for transmission between middle apical dendrite and soma when the soma is depolarized to −50 mV exhibits the typical downward shift relative to the profiles obtained when soma was at hyperpolarized/resting membrane potentials. In general, the lowest transfer resonance quality was observed to be in transmissions from basal dendrite to soma. These profiles exhibited the usual banding structure where the profiles obtained when soma was at hyperpolarized and resting potentials had the higher values of transfer resonance quality and were clustered much closer together. The transfer resonance quality between basal dendrite and soma peaked at 2.8 when both compartments were hyperpolarized, and when the soma was depolarized to −50 mV, transfer resonance between basal dendrite and soma nearly disappears.

Transfer resonant frequency for middle apical and basal dendrite compartments is shown in Figure 5B. As soma membrane potential was increased from hyperpolarized to depolarized values, the transfer resonant frequency became progressively faster for signals transmitted from both middle apical and basal dendritic compartments to the soma. The bands of resonant transfer frequency for middle apical dendrite-to-soma and basal dendrite-to-soma transmissions increased as dendritic membrane potential increased and were partially overlapping. For hyperpolarized, resting, and depolarized soma, the corresponding middle apical dendrite-to-soma bands were 5–15 Hz, 10–20 Hz, and 15–25 Hz, respectively. Similarly, for hyperpolarized, resting, and depolarized soma, the corresponding basal dendrite-to-soma bands were 5–11 Hz, 10–14 Hz, and 17–21 Hz.

Transfer resonant impedance for middle apical and basal dendrites is shown in Figure 5C. Like both distal apical compartments, basal dendrite and middle apical dendrite compartments had transfer resonant impedance profiles for the cases when the soma was at −80 and −65 mV that group together at much higher values than when the soma was at −50 mV. For basal dendrite-to-soma transmissions, the highest transfer resonant impedance values (40–45 MΩ) are observed for hyperpolarized basal dendrite compartment and hyperpolarized and resting soma. When basal dendrite is depolarized, the transfer resonant impedance decreases rapidly to values near 5 MΩ. When the soma is depolarized to −50 mV, the maximum transfer resonant impedance is 22 MΩ at hyperpolarized basal dendrite membrane potential and decreases to 3 MΩ when basal dendrite was depolarized to −30 mV. For middle apical dendrite-to-soma transmissions, the highest transfer resonant impedance values (25–28 MΩ) are observed for middle apical dendrite membrane potentials between −70 and −50 mV and hyperpolarized and resting soma. When middle apical dendrite is hyperpolarized or depolarized out of this range, the transfer resonant impedance decreases. When the soma is depolarized to −50 mV, the maximum transfer resonant impedance is 15 MΩ at hyperpolarized dendrite membrane potential and decreases to 3 MΩ when middle apical dendrite is depolarized to 0 mV.

### Resonance analysis on model without hot zone

#### Input resonance analysis

**Figure 6:**
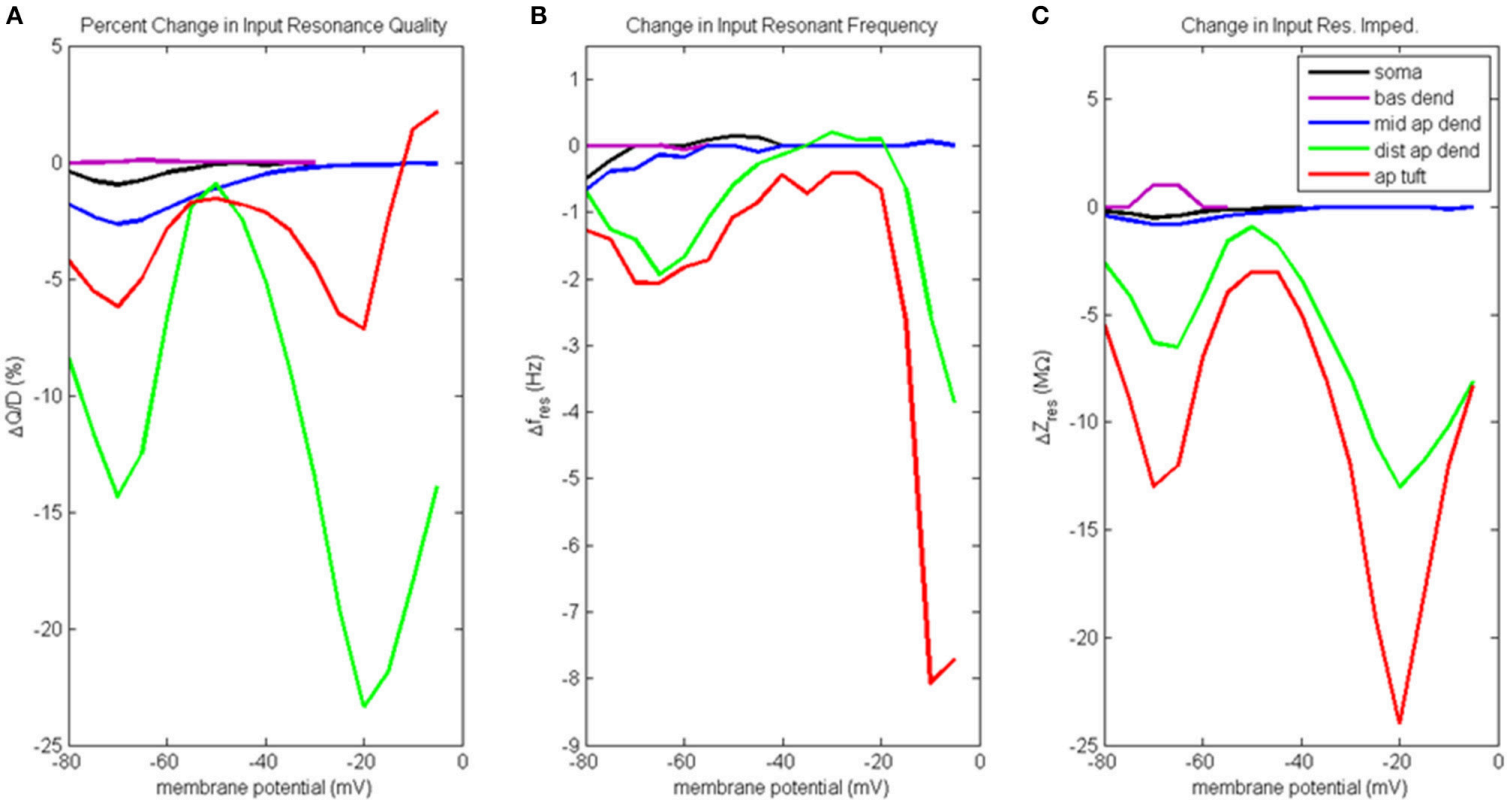
Change to input resonance analysis when hot zone is removed. **(A)** Percentage change to input resonance quality, **(B)** change to input resonant frequency, and **(C)** change to input resonant impedance, vs. initial compartment membrane potential.

A good illustration of changes to model behavior when the distal apical hot zone has been removed is to examine difference plots between the case with the hot zone and the case without the hot zone. Figure 6 illustrates how the outcome of our input resonance analysis changed when g_Ca(H)_ and g_Ca(L)_ density in distal apical dendrite and apical tuft is reduced. For profiles of input resonance quality, resonant frequency, and resonant impedance presented in the same format as Figure 3, and for the corresponding waveforms of compartment voltage response at key values of membrane potential, see Figures S5, S6, respectively.

Percentage change to input resonance quality when distal apical hot zone has been removed is shown in Figure 6A. Not surprisingly, there isn't much change to the resonance quality in the basal dendrite compartment. There was a small decrease in resonance quality for soma and middle apical dendrite at hyperpolarized/rest membrane potentials (1–2.5% decrease). On the other hand, much larger decreases to resonance quality was observed for the distal apical compartments where the g_Ca(H)_ and g_Ca(L)_ density was reduced. Two large decreases occur at membrane potentials of −70 and −20 mV. At −70 mV, resonance quality decreased by 6 and 14% for apical tuft and distal apical dendrite compartments, respectively. At −20 mV, resonance quality decreased by 7 and 23% for apical tuft and distal apical dendrite compartments, respectively.

Reducing g_Ca(H)_ and g_Ca(L)_ density in distal apical compartments also impacts HB values for the compartments during input resonance analysis (Table S2B). The soma and distal apical compartments all experienced a widening of HB relative to the results obtained on the baseline model, while middle apical dendrite and basal dendrite both experienced a modest narrowing of HB.

Figure 6B shows how each compartments' input resonant frequency changed when the hot zone was removed. Resonant frequency for the basal dendrite compartment does not change when distal g_Ca(H)_ and g_Ca(L)_ density is reduced. There is a small decrease to resonant frequency (<1 Hz) for soma and middle apical dendrite when they are at hyperpolarized membrane potentials. Larger decreases to resonant frequency are observed for distal apical compartments at hyperpolarized and very high levels of depolarization. Both distal apical dendrite and apical tuft experience up to 2 Hz reduction to resonant frequency at −65 mV, while at membrane potentials > −20 mV, the reduction experienced by distal apical dendrite and apical tuft is as much as 8 and 3.8 Hz, respectively.

Changes to input resonant impedance is shown in Figure 6C. With the reduction to distal g_Ca(H)_ and g_Ca(L)_ density, resonant impedance for the basal dendrite compartment increased by 1 MΩ at hyperpolarized membrane potentials. There was a small decrease to resonant impedance for soma and middle apical dendrite when they were at hyperpolarized membrane potentials (<1 MΩ). Like the case for resonance quality, the distal apical dendrite and apical tuft compartments show significant reductions in resonant impedance at −70 and −20 mV except this time, it is the apical tuft that was most affected by the reduced distal g_Ca(H)_ and g_Ca(L)_ density. At −70 mV, resonant impedance magnitude decreased by 6 and 13 MΩ for distal apical dendrite and apical tuft compartments, respectively. At −20 mV, resonant impedance magnitude decreased by 13 and 24 MΩ for distal apical dendrite and apical tuft compartments, respectively.

#### Transfer resonance analysis

**Figure 7:**
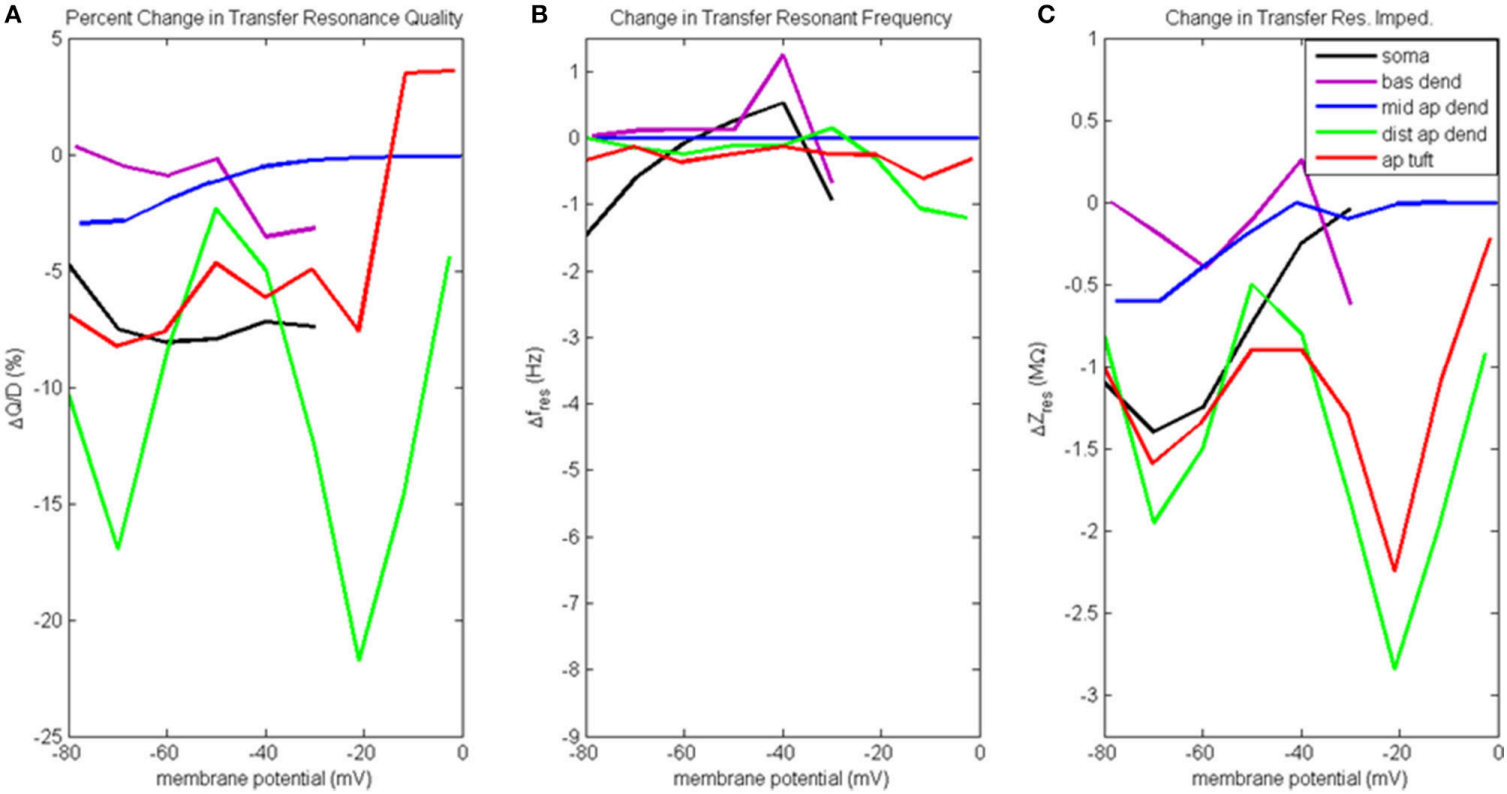
Change to transfer resonance analysis when hot zone is removed. **(A)** Percentage change to input resonance quality, **(B)** change to input resonant frequency, and **(C)** change to input resonant impedance, vs. initial compartment membrane potential (note the change in scale along the ordinate relative to Figure 6C).

In Figure 7 we again use difference plots to illustrate changes to model behavior when the distal apical hot zone has been removed. Profiles of transfer resonance quality, resonant frequency, and resonant impedance are presented in the same format as Figures 4, 5, and for the corresponding waveforms of compartment voltage response at key values of membrane potential, see Figures S7–S9, respectively. In the case of transfer resonance analysis performed on a model neuron without a distal apical hot zone, only simulations with the transfer compartment held near resting membrane potentials were performed (−60 mV for distal apical dendrite and −65 mV for soma).

Figure 7A presents the percentage change to transfer resonance quality. In general, when distal g_Ca(H)_ and g_Ca(L)_ density is reduced, there is a decrease in the transfer resonance quality between soma and distal apical dendrite, as well as between the dendritic compartments and the soma. Notably, the somatic transfer resonance quality decreased almost uniformly by 7–8%. In addition, the transfer resonance quality between distal apical dendrite and soma experienced reduced values at −70 mV (17% reduction) and −20 mV (22% reduction). In addition, the impact of removing the distal hot zone on HB values in the case of transfer resonance is not as clear as in the case of input resonance (Table S2D).

The change to transfer resonant frequency and transfer resonant impedance is presented in Figures 7B,C. For all compartments, both transfer resonant frequencies and transfer resonant impedance magnitudes changed very modestly in response to reduced distal g_Ca(H)_ and g_Ca(L)_ density. In the case of transfer resonant frequencies, the changes were no more than ±1.5 Hz. In the transfer resonant impedance profiles, the distal apical compartments showed reduced values at the same membrane potentials (−70 and −20 mV) at which reductions were observed in their input and transfer resonance quality, as well as input resonant impedance. In particular, the transfer resonant impedance of distal apical dendrite and apical tuft (as well as the soma) decreased by 1–2 MΩ when the compartment membrane potential was −70 mV. When membrane potential was −20 mV, transfer resonant impedance for distal apical dendrite and apical tuft decreased by 2–3 MΩ.

## Discussion

Using the concept of a distal “hot zone,” we have configured a model neocortical layer 5 pyramidal neuron to display enhanced coupling of perisomatic and distal apical spiking zones (Hay et al., 2011). Using this model, we applied both input and transfer resonance analysis in the soma and in several key locations within the dendritic tree to assess functionally-relevant differences in the response of different zones of the model. Furthermore, to gain insight about the role of distal g_Ca(H)_ and g_Ca(L)_ density in the coupling of perisomatic and distal apical regions, we performed the resonance analysis twice, once with the distal hot zone and once without.

### General pattern in resonance characteristics

At least four distinct regions of the model neuron can be distinguished based on the results of our resonance analysis. In describing these zones, we use the following convention for defining relevant brainwave bands (Buzsaki, 2011): theta (4–7 Hz), alpha (7–14 Hz), and beta (14–30 Hz).

Basal dendrite (and oblique dendrite, based on previous work not included in the current study) is very weakly resonant only at subthreshold membrane potentials and has input resonant frequencies that increase rapidly throughout alpha and beta range as membrane potential increases. It is more accurate to consider these compartments as having a very weak frequency preference spectrum at subthreshold membrane potentials and transitioning to a high-pass filter at suprathreshold potentials. Basal dendrite also had much higher input and transfer resonant impedance at near rest/hyperpolarized membrane potentials than any other compartment in the analysis. The high input and transfer impedance is partially due to the small size of this compartment and its close proximity to the soma, respectively.

The apical tuft has weak input resonance across the entire range of membrane potentials considered and has a flat input resonant frequency profile that is almost entirely confined to alpha frequencies. On the other hand, apical tuft has very strong transfer resonance with a flat transfer resonant frequency profile that varies depending on soma membrane potential—low alpha when soma hyperpolarized, high alpha when soma depolarized, in our simulations. The apical tuft had moderately high values of input and transfer resonant impedance across a very broad range of membrane potentials, particularly when membrane potential was > −50 mV.

The soma and middle apical dendrite can be said to belong to a perisomatic zone along the soma-apical dendrite axis. These compartments have very strong input resonance at hyperpolarized/resting membrane potentials, and their resonant frequency profiles increase from theta to mid beta as compartment membrane potential is increased. Both compartments had low overall input resonant impedance, but moderate to high transfer resonant impedance at resting and hyperpolarized membrane potentials.

Distal apical dendrite showed strong input resonance at moderate levels of depolarization, and a relatively flat input resonant frequency profile in low alpha range when membrane potential was < −50 mV, but an increasing profile from low alpha to mid beta when membrane potential was > −50 mV. Like apical tuft, distal apical dendrite also showed very strong transfer resonance with a relatively flat transfer resonant frequency profile that varies depending on soma membrane potential—theta/alpha border when soma hyperpolarized, alpha/beta border when soma depolarized. Distal apical dendrite had moderate values of resonant input and transfer impedance and the peak in both of these profiles occurs at depolarized membrane potentials—approximately −40 mV.

Because our model was configured to have a distal apical hot zone where g_Ca(H)_ and g_Ca(L)_ density is higher than the rest of the model, it is not surprising that resonance analysis is able to distinguish between perisomatic regions and distal apical regions. However, our results show that resonance analysis is also able to further divide the distal apical hot zone into two distinct zones, namely, distal apical dendrite and apical tuft. Our results indicate that the frequency preference of these distal regions of layer 5 pyramidal neurons becomes more specific at moderate to high levels of depolarization as evidenced by their input and transfer resonance quality scores, and their HB values. This increase in frequency preference is directly correlated to the impact of these distal apical regions on the soma as evidenced by their transfer resonant impedance. On the other hand, our results indicate that the frequency preference of proximal apical dendrite and soma is strongest at near resting and hyperpolarized membrane potentials (high input and transfer resonance quality, and small HB) and that, at least in the case of the soma, this is the condition under which signals transmitted to distal apical regions will have the highest transfer resonant impedance, and therefore, the largest functional impact on action potential generation. In short, the shifts along the soma-apical dendrite axis in maximum input resonance quality and flatness of input resonance quality profiles indicates that the further a compartment is from the soma on this axis, the weaker the frequency preference that it can attain, the more depolarized it needs to be to attain its peak frequency preference, and the weaker its dependence on a particular range of membrane potentials for resonance to occur. Furthermore, our results indicate that the basal dendrite (and presumably oblique dendrites) of layer 5 pyramidal neurons do not have strong frequency preferences. Their close proximity to the soma may not require the same level of specificity in frequency preference to have a large impact on the soma.

The four distinct zones identified in the current study are consistent with a three-layer model of layer 5 pyramidal neuron presented by Spruston and Kath (2004), which is based on synaptic integration and afferent connectivity throughout the neuronal membrane. The first layer of this model is an input layer comprising two distinct zones—perisomatic dendrites (basal and oblique), and apical tuft. The output of the first layer feeds into a second layer that acts as an integration layer. The second layer is comprised of two distinct integration zones—proximal apical dendrite and soma, and distal apical dendrite. The third layer is the action potential initiation zone in the axon hillock. The four zones identified in our resonance analysis correspond to the four zones used by Spruston and Kath (2004) to define the first two layers of their model layer 5 pyramidal neuron. In this view, layer 5 pyramidal neurons can be considered as having two input zones with weak input frequency preference, one close to the soma comprising basal and oblique dendrites, and one far from the soma in the apical tuft. However, significant frequency preference in signals transmitted from both of these input zones to the soma emerges due to the rather strong frequency preference displayed by the two integration zones of the neuron—distal apical dendrite and proximal apical dendrite/soma.

### Conditions for optimum subthreshold communication between soma and distal apical dendrite

Transfer resonance analysis also provides an indication of the conditions that should be most conducive to electrical coupling between the subthreshold perisomatic zone and the distal apical dendrite zone in pyramidal neurons. Communication between these two regions should be best when the soma is at hyperpolarized/resting membrane potentials while distal apical dendrite is depolarized to −40 mV and the signal encoding the information has a frequency of 7–12 Hz. Because of the shift in maximum input resonance quality along the soma-apical dendrite axis, the conditions for maximum electrical coupling between soma and distal apical dendrite also coincides with conditions for maximum frequency preference (input resonance quality) and response strength (input resonant impedance) to injected chirp currents for these two regions. In addition, there are key membrane potential values for which transfer resonant frequencies for some or all compartments converge onto the same value. For example, when the soma and distal apical regions are both hyperpolarized to −80 mV, the resonant transfer frequency for communication in both directions is 7.5 Hz (low alpha); when all compartments are near the resting membrane potential of the soma, −68 mV, they all have a transfer resonant frequency around 10 Hz (middle alpha); on the other hand, when the soma and distal apical regions are at −55 mV, they have a transfer resonant frequency of 15 Hz (low beta). It has been suggested that global coherence within dendritic oscillators plays a major role in the modulation of perisomatic spike generation (Remme et al., 2009). Our results suggest that a more homogeneous distributions of critical membrane potential values throughout the different regions of layer 5 pyramidal neurons may be associated with global coherence within these neurons.

It should be noted that when a DC offset is used to bring distal apical dendrite membrane potential to −40 mV, the somatic compartment remains near rest because it experiences very little of this distally-applied current. Therefore, situations when a layer 5 pyramidal neuron is at or below rest when it experiences input to its distal apical regions is sufficient to create the conditions for optimum modulation of somatic membrane potentials. Such a situation can arise when a quiescent period for a region of neocortex gives way to increased stimulation via input to distal regions of layer 5 pyramidal neurons, such as non-specific thalamocortical input or corticocortical feedback from distant neocortical areas (Spruston, 2008). If the input to the distal apical dendrite and apical tuft raises the local membrane potential to an average value of −40 mV while encoding an information signal at 7–12 Hz, it would maximize the subthreshold electrical coupling of distal apical dendrite and soma. During such conditions, oscillations in the soma will result from, and be phase-locked to, oscillations in distal apical dendrite, making it possible for perisomatic spike generation to be gated by distal apical synaptic inputs (Richardson et al., 2003; Remme et al., 2009).

Alternatively, the soma could receive its own modulating signal that would effectively tune its frequency preference for input to distal apical regions. For example, a slow sinusoidal modulating signal could cause the soma's membrane potential to oscillate between hyperpolarized potentials and some level of depolarized potential depending on the amplitude of the modulation. At the peaks and troughs of this modulation, the soma would be most responsive to distal apical input within distinct frequency ranges, such as low alpha at the troughs and mid-high alpha at the peaks. Somatic frequency preference to distal apical input would therefore be phase-locked to the signal modulating somatic membrane potential. This type of process has implications for the ways in which multi-frequency coupling could occur in the brain (VanRullen and Koch, 2003). In short, these results indicate that the interactions between different functional zones should be considered in a more complete understanding of neuronal integration. Resonance analysis, in particular transfer resonance, may, therefore be a useful tool for assessing the integration of inputs across the entire neuronal membrane.

### Calcium conductances [g_Ca(H)_ and g_Ca(L)_] amplify HCN and muscarinic resonance

The resonance observed in our model at hyperpolarized and resting membrane potentials is mediated by two currents: hyperpolarization-activated cyclic nucleotide-gated nonselective cation (HCN) and low-threshold calcium (Hutcheon et al., 1994, 1996; Hutcheon and Yarom, 2000; Ulrich, 2002). HCN (g_h_) generates resonance below about −60 mV, the strength of which is highly dependent on location due to the exponential gradient of increasing conductance density along the soma-apical dendrite axis (Narayanan and Johnston, 2008; Zhuchkova et al., 2013). Low-threshold calcium [g_Ca(L)_] generates a resonance within a narrow band of membrane potentials (approximately −80 to −65 mV) where the activation and inactivation functions overlap (Figure S10 Supplementary Materials). The inactivation curve generates the resonance while the activation curve amplifies it. The strength of the resonance amplified by g_Ca(L)_ in our model is also highly dependent on location due to the distal region of high-density for the Ca^2+^ ionic conductances.

Our results indicate that the high conductance density of g_Ca(L)_ present in the distal apical compartments in our model effectively amplifies subthreshold resonance, both in soma and distal apical regions, and increases the preferred input frequency of distal apical regions by up to 2 Hz in our model. The largest impact of g_Ca(L)_ occurs within the window current for this conductance, centered at −70 mV. This finding is consistent with the observation that g_Ca(L)_ and g_h_ act together to produce the slow depolarization that underlies burst firing in some neocortical pyramidal neurons that are excited from hyperpolarized/resting membrane potentials (Foehring and Wyler, 1990; Foehring and Waters, 1991).

Resonance in our model at depolarized membrane potentials is mediated by the muscarinic (K^+^) current, or M current [g_K(M)_]. For example, in the somatic compartment of our model, this current produces a voltage-dependent resonant frequency that varies from approximately 8 Hz at resting membrane potentials to 23 Hz at −40 mV (see Figure 3A, black line), which agrees well with the resonant characteristics attributed to the M current in neocortical pyramidal neurons (Gutfreund et al., 1995; Hutcheon and Yarom, 2000). A well-known amplifier of the g_K(M)_ resonance is the persistent sodium conductance [g_Na(P)_] (Hutcheon and Yarom, 2000). The reductions to input and transfer resonance quality, and input and transfer impedance at −20 mV observed in this study indicate that another important amplifier of g_K(M)_ resonance is the high-threshold calcium conductance [g_Ca(H)_]. This amplification peaks at −20 mV, the membrane potential at which g_Ca(H)_ is activating at the fastest rate (largest slope in activation function) and experiences its maximum time constant of 2.1 ms in our simulations (Figure S11 Supplementary Material).

The low membrane voltage (< −60 mV) resonance mediated by g_h_ and g_Ca(L)_ is strongest in distal apical regions where both conductances have high densities. On the other hand, the depolarized resonance (> −60 mV) mediated by g_K(M)_ and amplified by g_Na(P)_ and g_Ca(H)_ is strongest in the perisomatic region of the neuron (compartments 0–2 in Table S1), because this is where g_K(M)_ and g_Na(P)_ conductance densities are highest. This type of complementary resonance in the 3–12 Hz range has been observed in (and modeled for) CA1 hippocampal pyramidal neurons (Hu et al., 2009). The g_K(M)_ resonance in our study was observed to also extend into the range of beta frequencies (for example, see Figure 3A, black line), mostly due to the higher levels of membrane depolarization that we examine in this study. Extending the range of membrane potentials that our compartments were varied allowed us to identify the amplifying effect g_Ca(H)_ has on g_K(M)_ resonance. It has been noted before that g_Ca(H)_ has the kinetics to qualify it as an amplifier of resonance (Hutcheon and Yarom, 2000). Our results suggest a critical role for the interaction of g_Ca(H)_ with g_K(M)_ in the electrical coupling of distal apical and perisomatic regions of layer 5 pyramidal neurons—the amplification of g_K(M)_ resonance by g_Ca(H)_.

## Conclusion

We have shown that tuning of a neuron's post-synaptic physiological properties to enhance association between distant inputs across the neuronal membrane impacts resonance. Our results indicate that interactions between different functional zones need to be considered in a more complete understanding of neuronal integration and that resonance analysis may be a useful tool for assessing the integration of inputs across the entire neuronal membrane. The distinct zones that we have identified through resonance analysis are consistent with functional zones described by previous research, and the resonant interaction that we have observed between some of these zones has revealed new insights about the function of Ca^2+^ ionic conductances within layer 5 pyramidal neurons. By examining changes to resonance quality and resonant impedance when the distal Ca^2+^ hot zone is toggled, we showed that both g_Ca(H)_ and g_Ca(L)_ amplify resonance that is generated by two complementary conductances: g_h_ which becomes active below resting membrane potentials and is concentrated in distal apical regions, and g_K(M)_ which becomes active above resting membrane potentials and is concentrated in perisomatic regions. Reductions to both g_Ca(H)_ and g_Ca(L)_ densities in distal apical regions reduces amplification of these resonances and consequently, reduces the electrical coupling of distal apical and perisomatic regions of the neuron that is necessary for it to function as a coincidence detector for input to both of these regions. Natural next steps for this research include determining how resonance properties impact suprathreshold neuronal and network behavior.

## Author contributions

All authors contributed equally to experimental design and theoretical considerations. AY established single-neuron model and implemented all simulated current injection capabilities. MF ran all simulations and developed resonance analysis post-processing algorithms. All authors contributed to data interpretation and manuscript preparation.

### Conflict of interest statement

The authors declare that the research was conducted in the absence of any commercial or financial relationships that could be construed as a potential conflict of interest.
