# Supplementary material for: Resonance Analysis as a Tool for Characterizing Functional Division of Layer 5 Pyramidal Neurons
Source: Front Comput Neurosci. 2018 May 3;12:29. doi: 10.3389/fncom.2018.00029 (PMC5945999; doi:10.3389/fncom.2018.00029)
Supplement: Supplementary file 1 [file Data_Sheet_1.DOCX]

Supplementary Materials:

**Resonance Analysis as a Tool for Characterizing Functional Division of Layer 5 Pyramidal Neurons**

Melvin A Felton Jr^*^, Alfred B Yu, David L Boothe, Kelvin S Oie, Piotr J Franaszczuk

Correspondence:

Melvin A Felton Jr

[melvin.a.felton.civ@mail.mil](mailto:melvin.a.felton.civ@mail.mil)

Supplementary materials contains:

1 Supplementary model configuration

2 Supplementary resonance analysis

3 Supplementary results

4 Supplementary discussion

1 Supplementary model configuration

1.1 Conductance densities

The conductance density for each compartment in the baseline model is presented in Table S1. A general feature of this model is a high density of fast ionic conductances in perisomatic compartments and a high density of slow ionic conductances in distal apical compartments. This type of configuration has been shown to faithfully reproduce the spiking behavior of layer 5 pyramidal neurons (Mainen et al., 1996). Three of the ionic conductances whose densities are largest in perisomatic compartments include fast sodium (g_Na(F)_), persistent sodium (g_Na(P)_), and muscarinic potassium, or M current (g_K(M)_). The density in the initial axon compartment for g_Na(F)_ and g_K(M)_ was increased relative to Traub et al. 2003 to give the compartment properties of the initial axon segment known to be possessed by neocortical pyramidal neurons (Chung et al., 2006; Lai and Jan, 2006). In addition, g_Na(F)_ was increased in apical dendrite and tuft to facilitate back propagation of perisomatic spikes into distal apical regions (Gulledge and Stuart, 2003).

Three of the ionic conductances whose highest density values are in distal apical regions include high-threshold calcium (g_Ca(H)_), low-threshold calcium (g_Ca(L)_), and hyperpolarization-activated cyclic nucleotide-gated nonselective cation (HCN) (g_h_). The densities of these ionic conductances were patterned after values used by Hay et al. 2011. For instance, both calcium conductances were used to define a distal “hot zone”, comprising distal apical dendrite and apical tuft, where g_Ca(H)_ and g_Ca(L)_ had density values 10 and 100 times higher than anywhere else in the dendritic tree, respectively. In addition, values for g_(AR)_ along the soma-apical dendrite axis were defined using the same exponential function used by Hay et al. 2011.

The rest of the ionic conductances included in our model are potassium conductances—transient inactivating (g_K(A)_), delayed rectifier (g_K(DR)_), slowly activating and inactivating (g_K2_), and fast voltage- and Ca^2+^- dependent (g_K(C)_). Dendritic g_K(A)_ values were given a homogeneous distribution throughout the dendritic tree in accord with observations of layer 5 pyramidal neurons (Spruston 2008). Values for g_K(DR)_ were adjusted to achieve somatic action potential spikes with amplitudes up to +18 mV in response to 3 nA, 5 ms square wave current injection into the soma. Values for g_K2_ and g_K(C)_, particularly throughout apical dendrite and apical tuft, were chosen to best fit the behavior of distal apical regions of layer 5 pyramidal neurons to an EPSP-like current injection (Hay et al., 2011; Larkum, 2013). A Ca^2+^ dependent K^+^ afterhyperpolarization current used by Traub et al. 2003 and 2005 was excluded from our model. This type of current is involved in the control of repetitive firing (Mainen et al., 1996). The impact of this current on our model runs was negligible since we were primarily interested in the spiking behavior of the model to very brief current injections (5 ms) that do not induce repetitive firing, and our resonance analysis was performed on a model neuron that was incapable of producing action potentials due to inactivation of the fast sodium conductance (g_Na(F)_) which was achieved by setting the conductance density for this channel in every compartment to zero.

Table S1 Membrane conductance densities for baseline model layer 5 pyramidal neuron (mS/m^2^), by level in model structure

| Level | g_Na(F)_ | g_Na(P)_ | g_K(DR)_ | g_K(C)_ | g_K(A)_ | g_K(M)_ | g_K2_ | g_Ca(H)_ | g_Ca(L)_ | g_h_ | |
| --- | --- | --- | --- | --- | --- | --- | --- | --- | --- | --- | --- |
| 0 | 9000 | 5 | 450 | 0 | 6 | 600 | 15 | 0 | 0 | 0 | |
| 1 | 4500 | 5 | 450 | 0 | 6 | 300 | 15 | 0 | 0 | 0 | |
| 2 | 5550 | 5 | 750 | 288 | 200 | 250 | 15 | 6 | 30 | 1 | |
| 3 | 750 | 0.6 | 250 | 288 | 40 | 136 | 0 | 0.3 | 0.5 | 0.5 | 1.76 |
| 4 | 150 | 0.12 | 0 | 9 | 40 | 136 | 0 | 1 | 5 | 0.5 | 2.45 |
| 5 | 150 | 0.12 | 0 | 9 | 40 | 136 | 0 | 1 | 5 | 0.5 | 3.32 |
| 6 | 1500 | 1.2 | 500 | 288 | 40 | 136 | 15 | 0.3 | 0.5 | 1.7635 | |
| 7 | 750 | 0.6 | 150 | 288 | 40 | 136 | 15 | 0.3 | 0.5 | 2.4515 | |
| 8 | 165 | 0.12 | 2 | 9 | 40 | 136 | 15 | 0.3 | 0.5 | 3.3194 | |
| 9 | 165 | 0.12 | 2 | 9 | 40 | 40 | 0 | 0.3 | 0.5 | 4.4141 | |
| 10 | 165 | 0.12 | 2 | 9 | 40 | 40 | 0 | 0.3 | 0.5 | 5.7950 | |
| 11 | 165 | 0.12 | 2 | 9 | 40 | 40 | 0 | 0.3 | 0.5 | 7.5368 | |
| 12 | 165 | 0.12 | 2 | 9 | 40 | 40 | 0 | 0.3 | 0.5 | 9.7338 | |
| 13 | 165 | 0.12 | 2 | 9 | 40 | 40 | 0 | 0.3 | 0.5 | 12.5050 | |
| 14 | 165 | 0.12 | 2 | 9 | 40 | 40 | 0 | 0.3 | 0.5 | 16.0006 | |
| 15 | 165 | 0.024 | 2 | 9 | 40 | 40 | 0 | 0.3 | 0.5 | 20.4098 | |
| 16 | 165 | 0.024 | 2 | 9 | 40 | 10 | 10 | 3 | 50 | 25.9714 | |
| 17 | 165 | 0.024 | 2 | 9 | 40 | 10 | 10 | 3 | 50 | 32.9866 | |
| 18 | 165 | 0.024 | 2 | 9 | 40 | 10 | 10 | 3 | 50 | 41.8355 | |
| 19 | 165 | 0.024 | 2 | 9 | 40 | 10 | 10 | 3 | 50 | 52.9971 | |

Explanation of levels: level 0 = initial axon; level 1 = rest of axon; level 2 = soma; level 3 = proximal basal and oblique dendrites; level 4 = middle basal and oblique dendrites; level 5 = distal basal and oblique dendrites; levels 6-18 = progressively more distal apical dendrite shaft; level 19 = apical tuft. Columns in maroon are the conductances patterned after Hay et al. 2011. The Ca^2+^ conductances have densities that are 1–2 orders of magnitude higher in distal apical dendrite and apical tuft than anywhere else in dendritic tree. The HCN conductance densities use the same exponential function as Hay et al. 2011. Left and right entries in HCN for levels 3–5 correspond to basal and oblique dendrites, respectively.

1.2 Conductance kinetics

All conductance kinetics, including reversal potentials, were left the same as in Traub et al. 2003 and Traub et al. 2005 with the exception of the M current whose activation curve was shifted by up to 15 mV in the hyperpolarized direction near the base of the activation curve, and whose time constant maximum value was reduced by 40 ms (see Equations S1-S2 and Figure S1). Changing the M current in this way allows it to become active at subthreshold membrane potentials, which more accurately models the resonance characteristics attributed to the M current of neocortical pyramidal neurons when their membrane potential is anywhere from rest to depolarized by about 30 mV (Gutfreund et al., 1995; Hutcheon and Yarom, 2000).

$$\alpha= \frac{0.02}{1+exp[111.11 \left( -V-0.022 \right)]}, \beta=0.01\exp\left[ 55.56\left( -V-0.043 \right) \right] (S1)$$

$$activation= \frac{\alpha}{\alpha+ \beta}, time constant= \frac{0.001}{\alpha+ \beta} (S2)$$

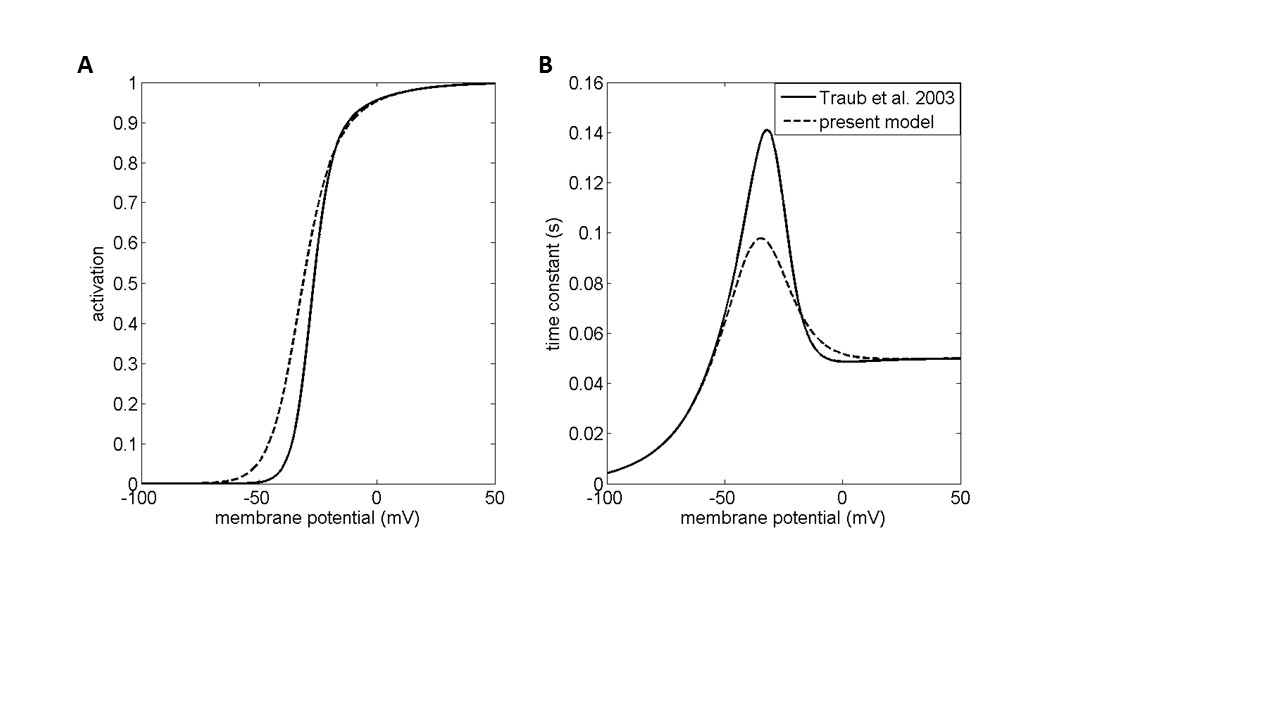


Figure S1 (A) Activation function for Traub et al. 2003 M current (solid) and the M current used in the present study (dashed). (B) Time constant function for Traub et al. 2003 M current (solid) and the M current used in the present study (dashed).

1.3 Passive electronic properties

Passive electrical parameters for the soma and all dendritic compartments were: membrane resistivity, R_m_= 5 Ω-m^2^; internal resistivity, R_i_= 1.5 Ω-m^2^; and membrane capacitance density, C_m_= 0.01 F/m^2^. The passive electrical parameters for the axon were: R_m_= 0.1 Ω-m^2^; R_i_= 1.0 Ω-m^2^; and C_m_= 0.009 F/m^2^. The leak reversal potential for the model was -90 mV. The temporal resolution of the simulations was 10^-4^ s.

- 1. EPSP-like current injection to distal apical dendrite compartment

$$dist ap dend I_{inject}= -{3.07*10}^{-9}[\exp\left( \frac{-t}{0.002} \right)-\exp\left( \frac{-t}{0.005} \right)] (S3)$$

2. Supplementary resonance analysis


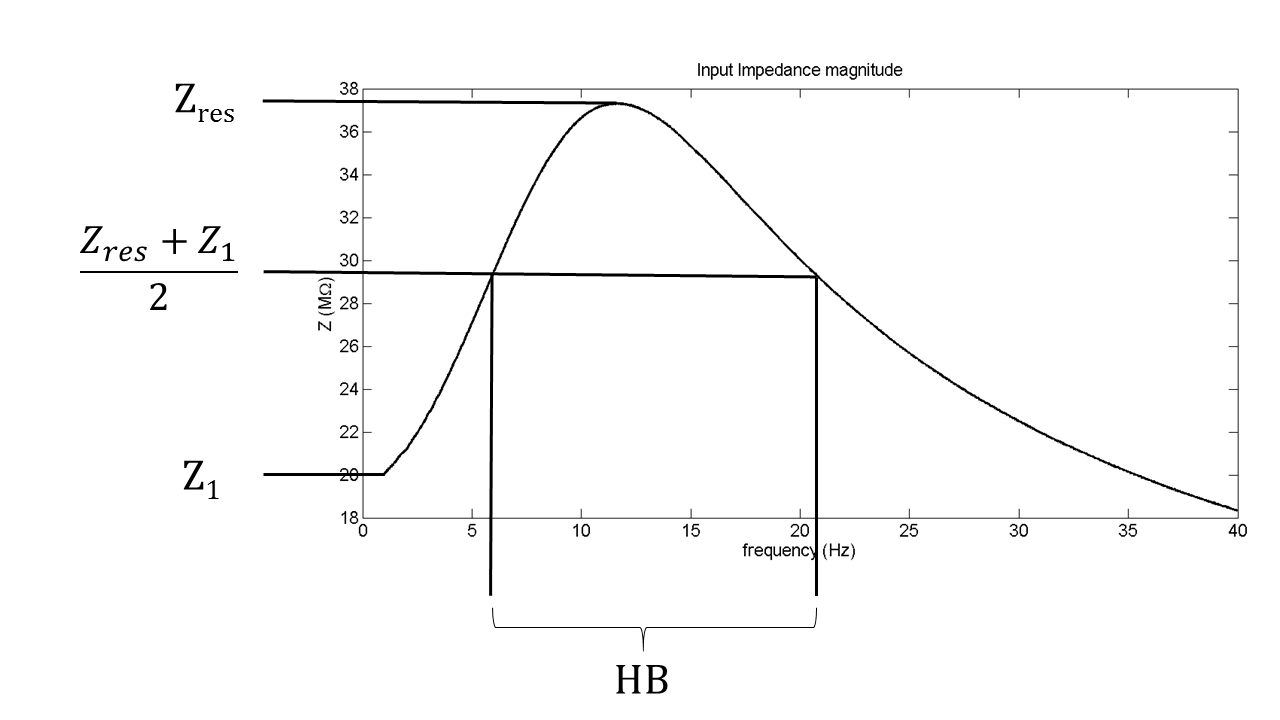


Figure S2 Illustration of half bandwidth (HB) calculation.

1. Supplementary results


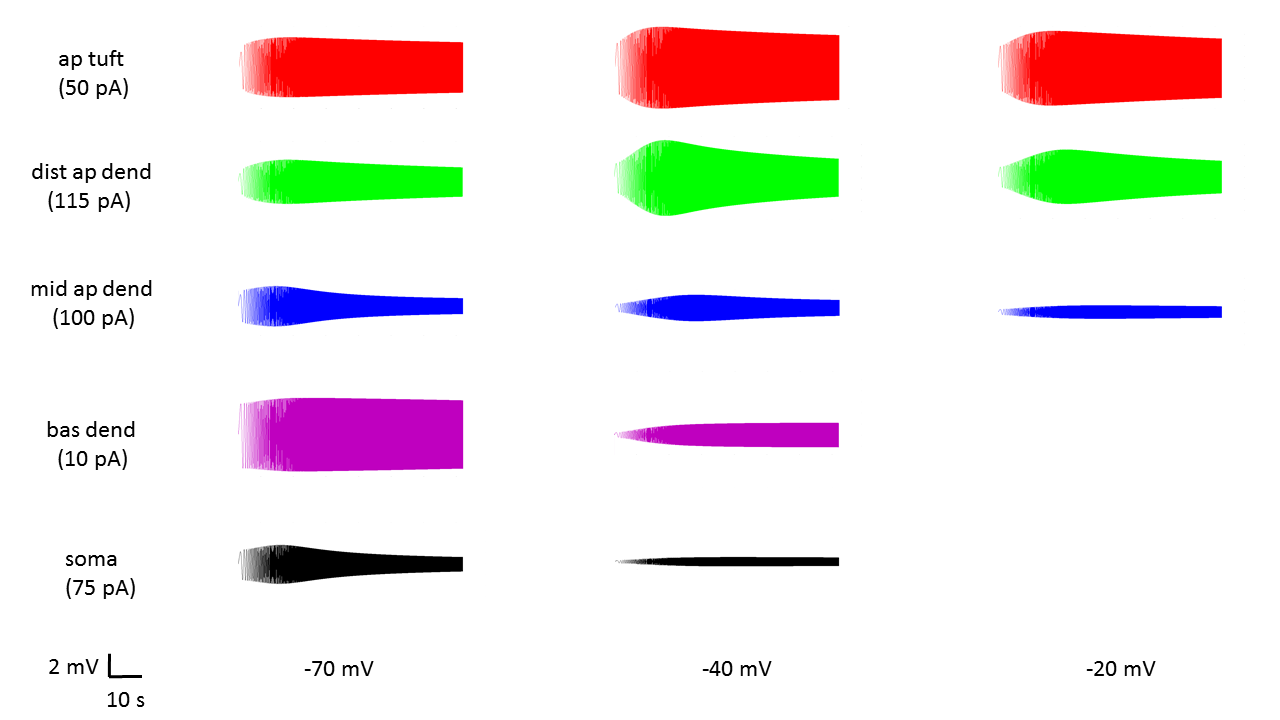


Figure S3 Membrane potential oscillations at -70 mV, -40 mV, and -20 mV for the compartments used to perform input resonance analysis on model with hot zone. Chirp function amplitude given in parentheses below compartment name on the left. Scale bar lower left.


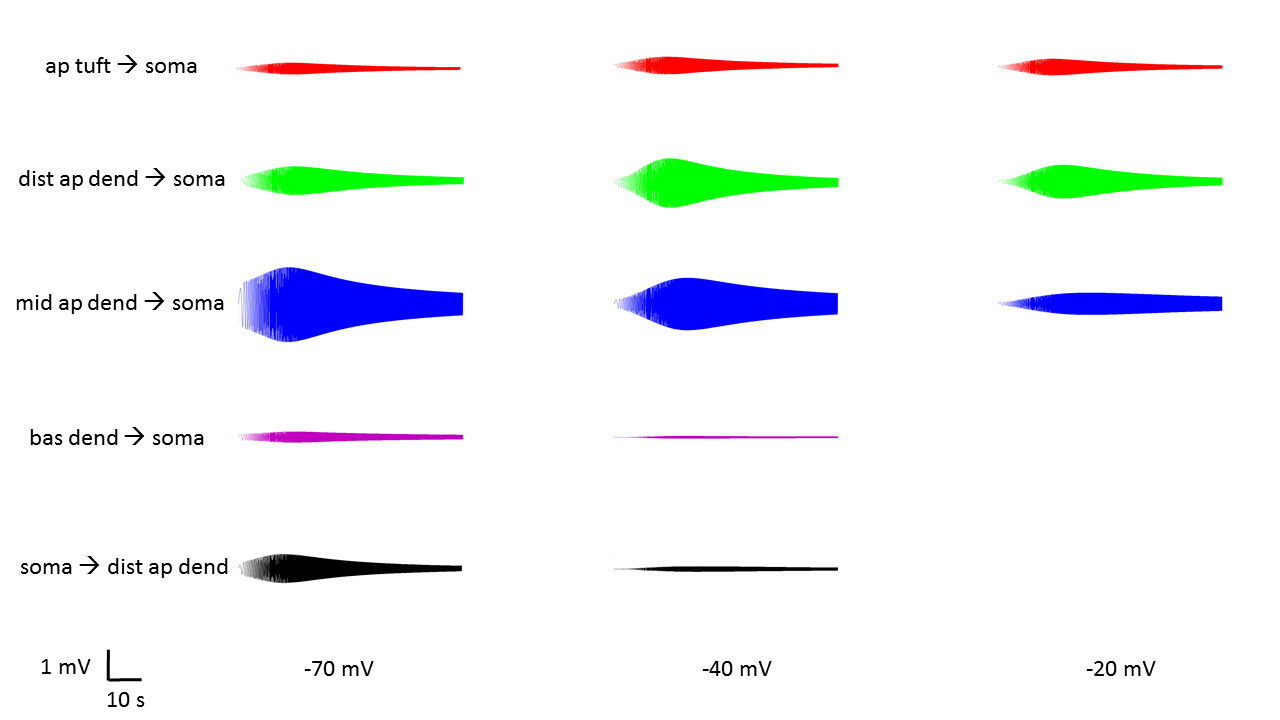


Figure S4 Membrane potential oscillations at -70 mV, -40 mV, and -20 mV for the compartments used to perform transfer resonance analysis on model with hot zone. Transfer direction indicated on the left. Scale bar lower left.


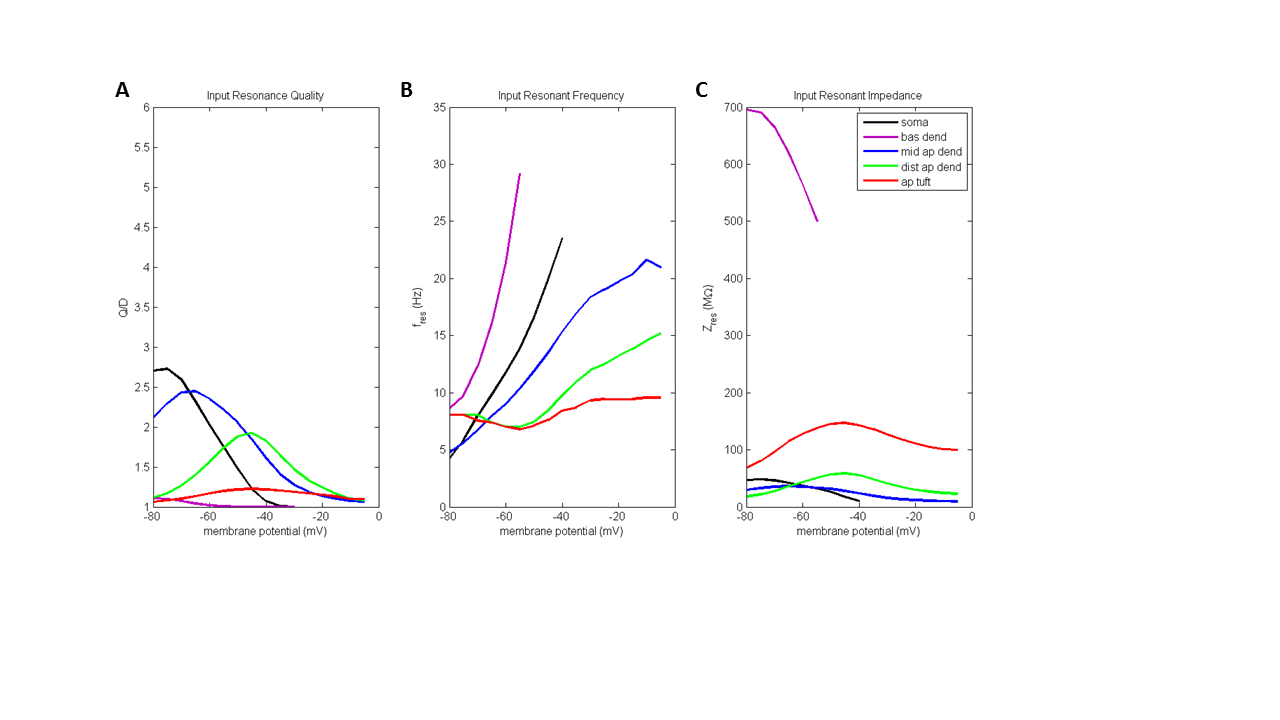


Figure S5 Input resonance analysis performed on model neuron without hot zone. (A) Input resonance quality (Q/D), (B) input resonant frequency, and (C) Input resonant impedance, versus initial compartment membrane potential.


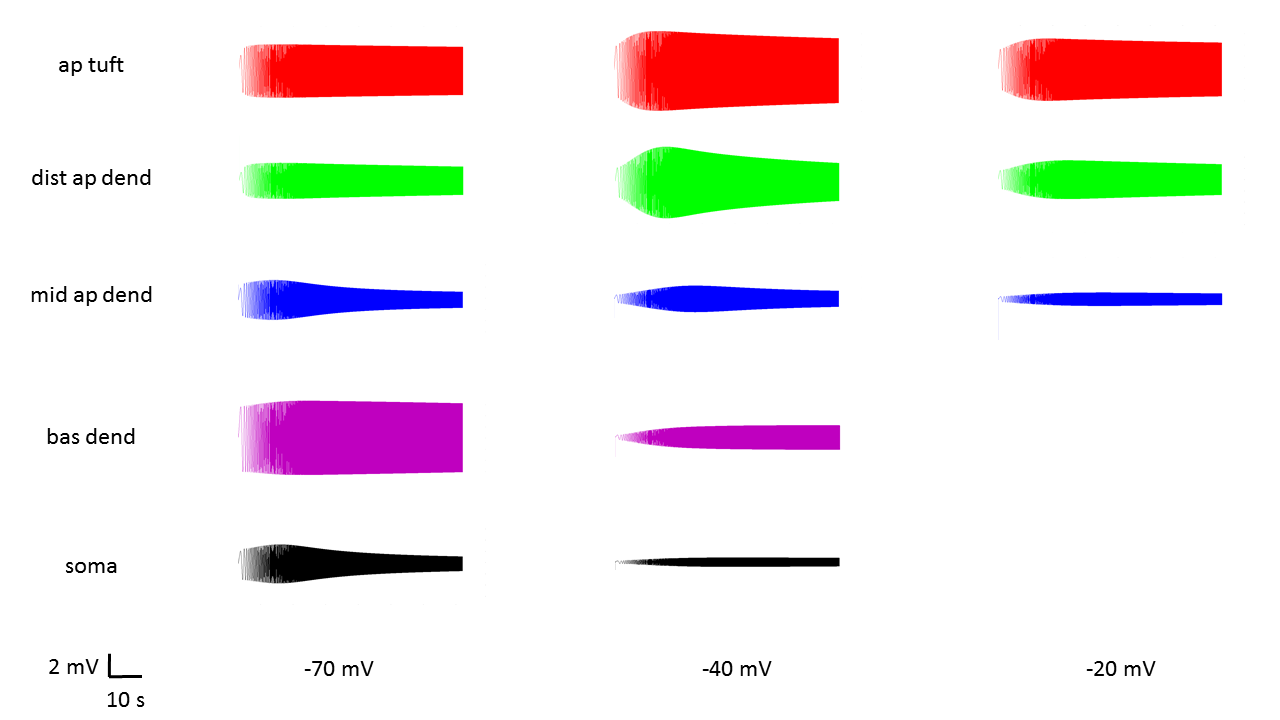


Figure S6 Membrane potential oscillations at -70 mV, -40 mV, and -20 mV for the compartments used to perform input resonance analysis on model without hot zone. Scale bar lower left.


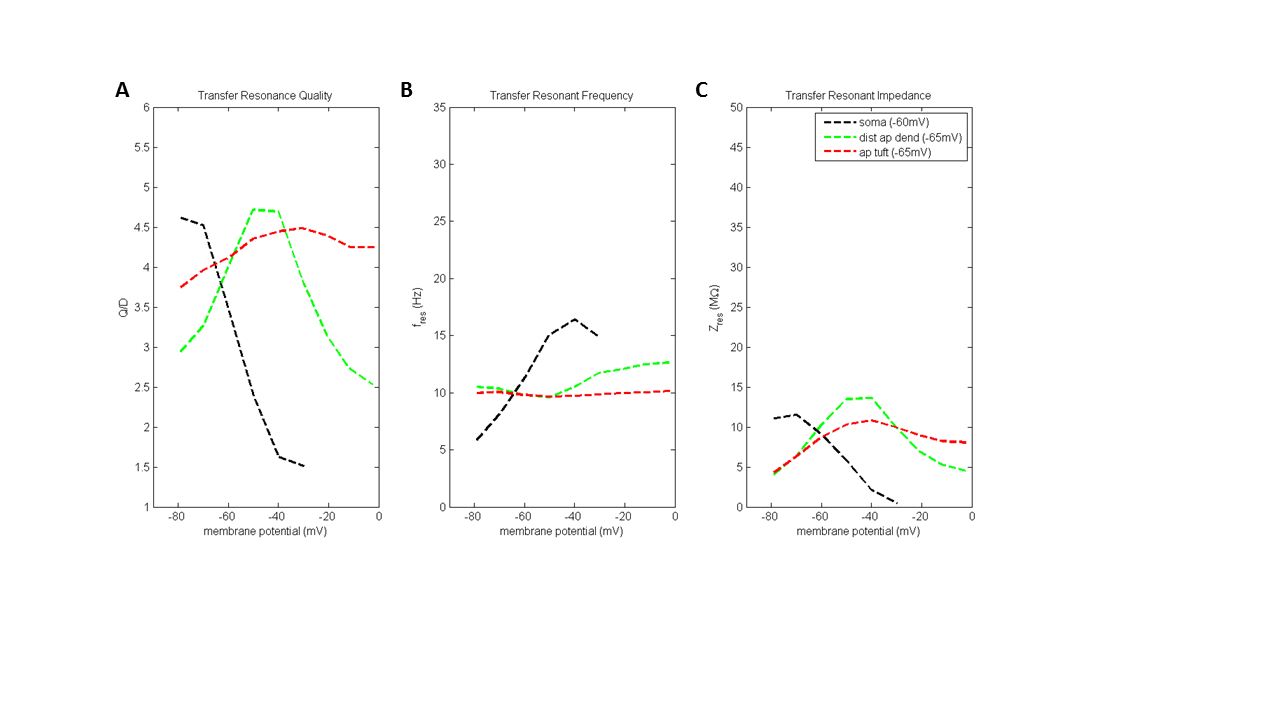


Figure S7 Transfer resonance analysis for model neuron without hot zone (soma, distal apical dendrite, apical tuft). (A) Transfer resonance quality (Q/D), (B) transfer resonant frequency, and (C) transfer resonant impedance, versus initial compartment membrane potential. In the legend, the membrane potential of the transfer compartment is given in parentheses next to the name of the injection compartment.


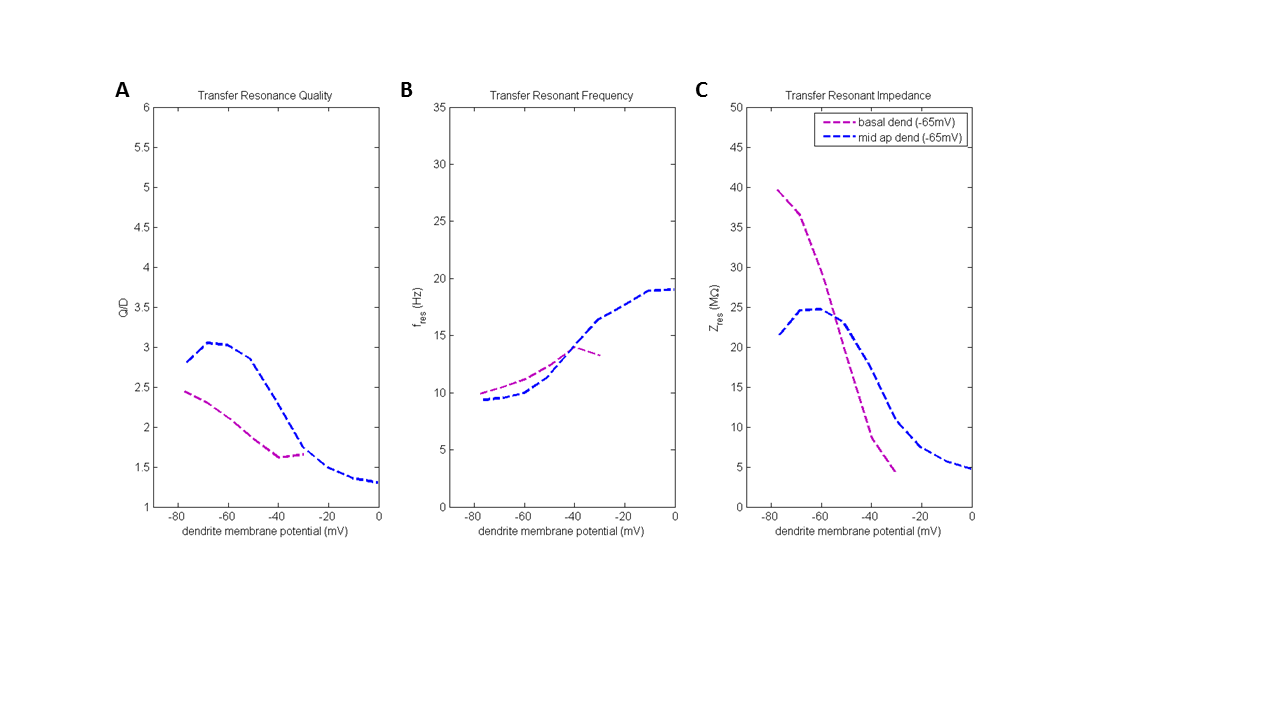


Figure S8 Transfer resonance analysis for model neuron without hot zone (basal dendrite and middle apical dendrite). (A) Transfer resonance quality (Q/D), (B) transfer resonant frequency, and (C) transfer resonant impedance, versus initial compartment membrane potential. In the legend, the membrane potential of the transfer compartment is given in parentheses next to the name of the injection compartment.


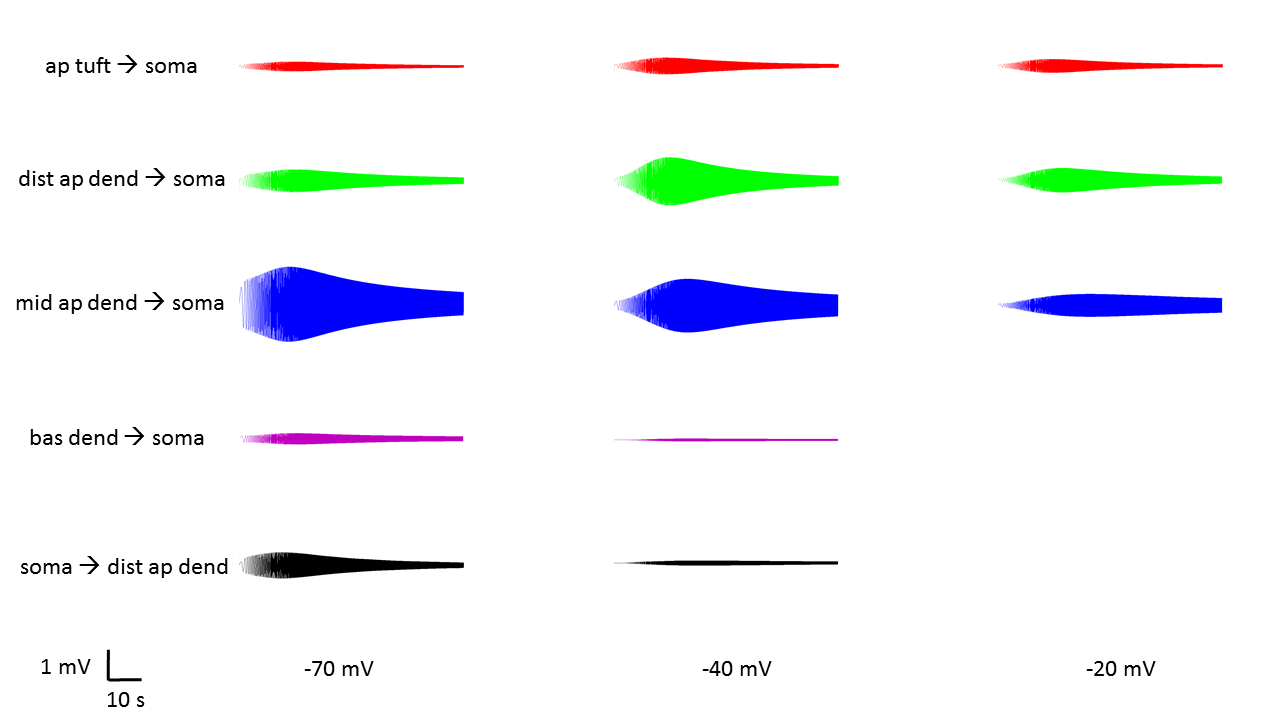


Figure S9 Membrane potential oscillations at -70 mV, -40 mV, and -20 mV for the compartments used to perform transfer resonance analysis on model without hot zone. Transfer direction indicated on the left. Scale bar lower left.


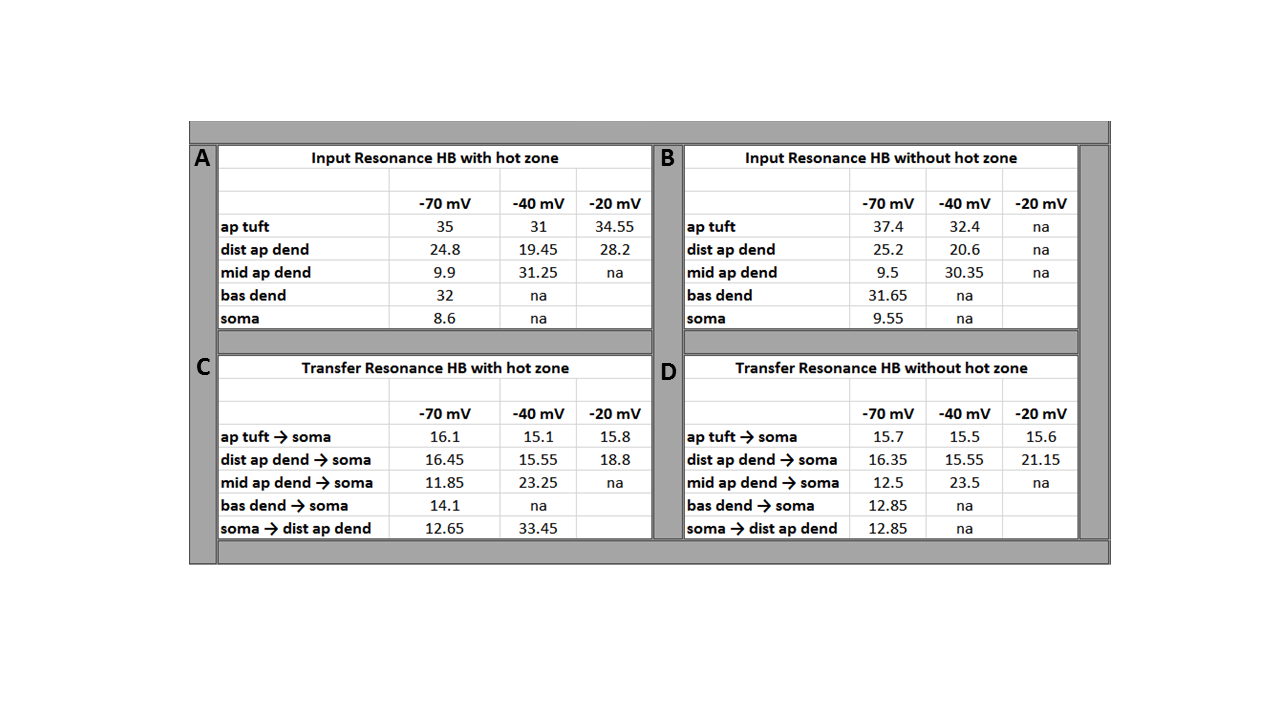


Table S2 HB (in Hz) calculated for the scenarios presented in Figures S3, S4, S6, and S9. (A) HB for Figure S4. (B) HB for Figure S5. (C) HB for Figure S6. (D) HB for Figure S7. Note that the resonance analysis for basal dendrite and soma was performed on the -80 mV to -30 mV range. Therefore, there are no entries for -20 mV for these compartments. An entry of “na” indicates that HB is so wide that it is not well-defined on the interval 1 Hz to 40 Hz.

4. Supplementary discussion


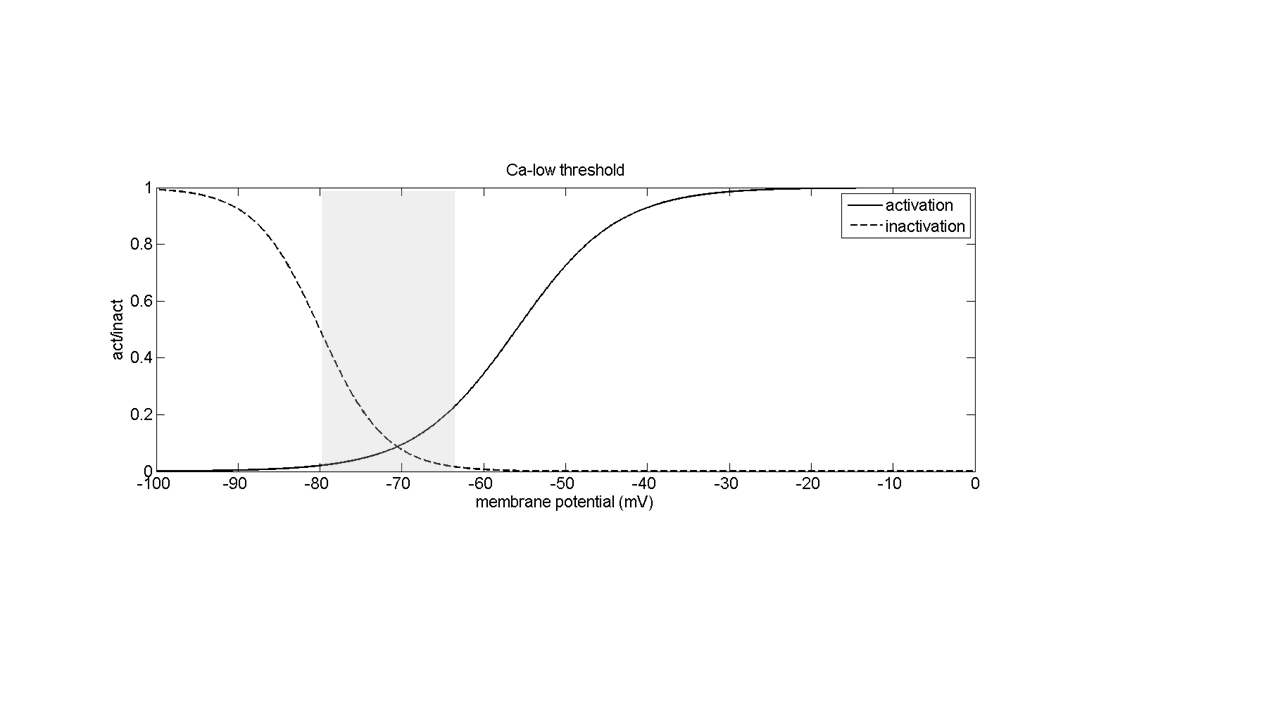


Figure S10 Activation and inactivation functions for low-threshold calcium conductance. The inactivation function generates a low frequency resonance that is amplified by the activation function. This resonant feature occurs due to a “window” current established by the overlap of the activation and inactivation functions, centered at -70 mV (shaded region).


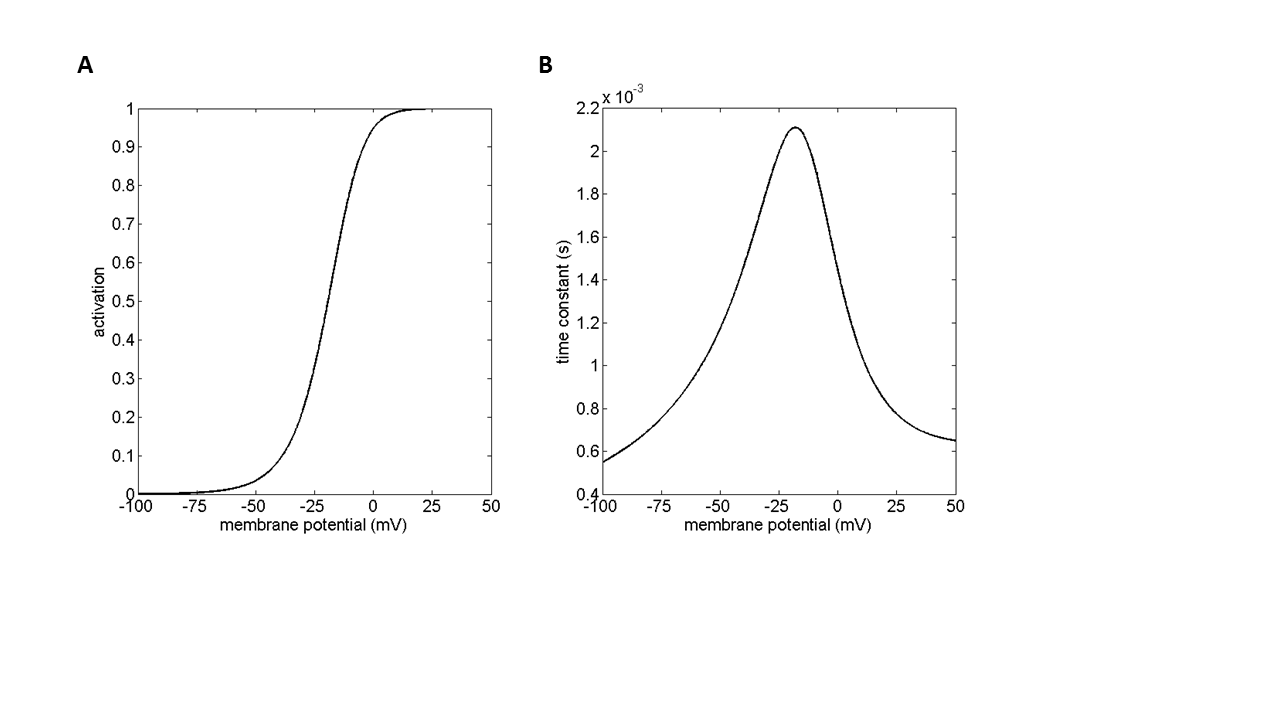


Figure S11 (A) High-threshold calcium conductance activation function. (B) High-threshold calcium conductance time constant.
